# Salt processing: A unique and classic technology for Chinese medicine processing

**DOI:** 10.3389/fphar.2023.1116047

**Published:** 2023-01-30

**Authors:** Ajiao Hou, Jiahao Lv, Shihao Zhang, Jiaxu Zhang, Liu Yang, Hai Jiang, Haixue Kuang

**Affiliations:** Key Laboratory of Basic and Application Research of Beiyao, Heilongjiang University of Chinese Medicine, Ministry of Education, Harbin, China

**Keywords:** salt processing, traditional Chinese medicine, chemical composition, pharmacodynamic effect, *in vivo* behavior

## Abstract

Traditional Chinese medicine (TCM) processing is the summary of long-term clinical experience, processing can change the property, taste and meridian of TCM, achieve the purpose of reducing toxicity and increasing efficiency, to ensure the safety of clinical medication. This paper summarizes the research advance of salt processing in recent years from the aspects of the types of excipients, salt processing methods, salt processing purpose and the influence of salt process on the chemical composition, pharmacodynamic effect and *in vivo* behavior of TCM, and analyzes and discusses the shortcomings of the current research, so as to provide ideas for the further research and development of salt processing of TCM. The literatures were classified and summarized by consulting the scientific database (such as SciFinder Scholar, CNKI, Google Scholar, Baidu Scholar, etc*.*), Chinese herbal classics and Chinese Pharmacopoeia and so on. The results show that salt processing is helpful to introduce drugs into the kidney channel and enhance the effect of nourishing Yin and falling fire. The pharmacological effect, chemical composition and *in vivo* behavior of TCM will change after salt processing. In the future, we should strengthen the research on standardizing the dosage of excipients, the quality standard after processing, the relationship between the change of chemical composition after salt processing and the enhancement of pharmacological efficacy, so as to deeply explain the principle of salt processing, and further improve and optimize the salt making process. By combing the influence of the of salt processing of TCM and analyzed the current problems, we hope to provide guidance for the in-depth study of the salt processing mechanism of TCM and the inheritance and innovation of TCM processing technology.

## Introduction

TCM is a kind of medicine applied to prevent and treat diseases under the guidance of TCM theory. Chinese medicinal materials can not be directly used in clinic, but must be processed into medicinal slices under the guidance of Chinese medicine processing theory before they can be used in clinical prescription compatibility. For example, some TCMs have certain toxicity, and if they are used directly, clinical safety cannot be guaranteed. In addition, some TCMs cannot exert their due efficacy after simple decoction. Therefore, how to take effective measures to reduce the toxicity and improve the efficacy of TCM has become a common focus of clinical medical workers.

Processing can change the property, taste and meridian of TCM, achieve the purpose of reducing toxicity and increasing efficiency, to ensure the safety and effectiveness of clinical medication. This is not only the characteristic of clinical use of TCM, but also a major feature of TCM, also one of the significant signs that TCM is different from other drugs. Salt processing method is a typical processing method of TCM. The traditional pharmaceutical process of processing drugs with salt as an auxiliary material is collectively referred to as salt processing method. Salt is first described in “*Leigong Treatise on the Preparation (雷公炮炙论)*”, has the effect of detoxification, cooling blood, moistening dryness, calming pain and relieving itching (Zhang and Wang, 2003). Modern medicine believes that salt mainly contains NaCl and trace amounts of MgCl_2_, CaCl_2_, KCl, NaI and other substances, among which NaCl is essential for maintaining normal osmotic pressure of human tissues. Entering into the stomach can promote gastric juice secretion and protein absorption, and can enhance urinary function and diuretic effect. There are more than 100 kinds of TCMs processed with salt in the past dynasties, and dozens of salt processing methods are clearly described, which can be divided into salt washing, salt infiltration, salt infiltration heating and salt stir-frying method (Zhou et al., 2021). It is mainly used for tonifying kidney and essence, treating hernia, relieving pain, diuresis and falling fire. It has the effect of inducing medicine downward, enhancing the curative effect, alleviating the dryness of medicine, strengthening the function of nourishing Yin and falling fire.

Salt processing method has a long history. Since the 1960s, there have been reports on the research of salt processing of TCM (Si and Zhao, 2019). However, at present, the prominent problem in the research of salt processing method is that the basic research is relatively weak, the processing mechanism of most TCM is not clear, and few reports on the comprehensive analysis of salt processing. Therefore, in order to further clarify the salt processing principle and improve the salt making process, this study comprehensively evaluates and analyzes the contents of the salt processing research of TCM in recent years, including the salt processing historical evolution, salt types, salt processing methods, salt processing purpose, the influence of salt processing on the chemical composition, pharmacodynamic effect and *in vivo* behavior, and the problems existing in the salt processing research, in order to provide reference for the further research and development of salt processing and the clinical use of TCM.

## Type of salt

Salt is not only a necessity of life, but also one of traditional Chinese medicine. The traditional pharmaceutical technology, which uses it as an excipient to process desicines, is collectively referred to as salt processing method, which is one of the important contents of the processing of TCM excipients. In order to better understand the intention of salt processing by predecessors, inherit the traditional salt processing technology and analyze the mechanism of salt processing, the types of salt are summarized (Wang et al., 2017; Li et al., 2018).

Before the Qing Dynasty, the varieties of salt were recorded in books, and different salts were used in processing. In “*Compendium of Materia Medica (本草纲目)*” Shizhen Li divided the varieties of salt into three categories: salt, Qing salt and Bai salt:

Salt, derived from a place where salt water can be stored, such as seawater, crystallizes after boiling and drying (Zhao et al., 2006). The use of salt as an auxiliary material for processing TCM was first seen in “*Leigong Treatise on the Preparation*”, such as Ricini Semen, the dried ripe seed of the *Ricinus communis* subsp. *Leucocarpus* (Bertol.) Müll.Arg. “ if use, boil it in salt soup for half a day”; Ostreae Concha, the shell of *Ostrea gigas* Thunberg, *Ostrea talienwhanensis* Crosse or *Ostrea rivularis* Gould “need to be boiled with a certain amount of salt for a day and a night”. Salt was the most commonly used in salt processing in the past dynasties. In Song and Ming Dynasties, salt processing TCMs and methods were rapidly increased and improved. At present, the Chinese Pharmacopoeia (2020 edition), the National Processing Standards of Traditional Chinese Medicine and the Processing Standards of All Provinces and Autonomous Regions stipulate that salt excipients are all salt. Salt has been used since ancient time (Huang et al., 2007; Si and Zhao, 2019).

Qing salt is derived from the crystal of halite (Zhao et al., 2006). The processing of TCM with Qing salt as processing excipients originally came from the “*Peaceful Holy Benevolent Prescriptions (太平圣惠方)*” of the Song Dynasty: Chaenomelis Fructus, the dried near ripe fruit of *Chaenomeles speciosa* (Sweet) Nakai, peel and pulp, and process it with sulfur and blue salt. Qing salt processed medicine was used in ancient times, but it is no longer used in modern times (Huang et al., 2007; Si and Zhao, 2019).

Bai salt, source of chloride stone salt group mineral stone Halite colorless transparent crystal, chemical composition is the same as Qing salt, less impurities. The use of Bai salt in the processing of medicine was first seen in “*Leigong Treatise on the Preparation*”: Citri Reticulatae Pericarpium Viride, the dried near ripe fruit of *Citrus reticulata* Blanco, needs to be processed with white salt. Bai salt processing medicine is rarely used in the past dynasties, and is no longer used in modern times (Huang et al., 2007; Si and Zhao, 2019).

“*Essence on the Silvery Sea (银海精微)*” in Tang Dynasty recorded that Qing salt was used in processing Aconiti Lateralis Radix Praeparata (the processed product of the child root of *Aconitum carmichaelii* Debx.). “*Empirical Recipes from Auspicious Bamboo Hall (瑞竹堂经验方)*” in Yuan Dynasty recorded that Qing salt and salt were simultaneous used to process Atractylodes Rhizoma (the dried roots of *Atractylodes lancea* (Thunb.) DC.), indicating that Qing salt and salt were used as two different excipients. “*Prescriptions for Universal Relief (普济方)*” in Ming Dynasty recorded that when salt processing Zingiberis Rhizoma Recens (the fresh rhizome of *Zingiber officinale* Roscoe), if there was no Qing salt, salt was used instead. After that, there was a tendency for the varieties of salt to merge. Most of the salt used in salt processing was not specified in books, simply recorded as salt, and some books clearly recorded salt was used in salt processing (Li et al., 2018). With the gradual maturation of salt production technology, at present, the clinical is mainly processed TCM with salt (Xu, 2018). Salt contains more than 97% NaCl and a small amount of MgCl_2_, MgSO_4_ and CaSO_4_ (Zhou et al., 2021). It probably associated with the fact that salt is more widely available. However, the amount of salt processing excipients is rarely recorded.

## Salt processing methods

Salt as processing excipients is an important part of its application in TCM. With the continuous improvement of the theory of TCM, the salt processing methods of all dynasties have been enriching, and the salt processing herbs have become increasingly complex and diverse. Salt processing beginning in “*Leigong Treatise on the Preparation*” in the southern and northern Dynasties, which recorded six kinds of TCMs processing with salt. In the Sui Dynasty, the salt processing method for the toxic herbal Aconiti Radix (the dried mother root of *Aconitum carmichaelii* Debx.) appeared. In Song Dynasty, the salt processing methods and varieties of TCM gradually increased. The Ming Dynasty recorded the most types and methods of salt products in history, among which “ *Prescriptions for Universal Relief* ”, “*Concise Herbal Foundation Compilation (本草品汇精要)*” and “*Compendium of Materia Medica*” made great contributions. After the Ming Dynasty, the methods and varieties of salt production were reduced, and after the Qing Dynasty, the previous salt processing method was used (Si and Zhao, 2019). This may be related to the following factors: (1) One of the main purposes of salt processing in ancient times was preservation and corrosion protection, but now it is almost unnecessary to use salt for preservation processing in producing areas, (2) In ancient times, the lack of materials and diseases may be related to the general lack of salt, but now salt is very common, and the nature of diseases has changed (Li et al., 2018). It is reported that there are dozens of methods for salt processing methods in history. Some methods are similar and can be divided into four categories: salt washing, salt infiltration, salt infiltration heating and salt stir-frying method. According to the different heating methods, saltwater infiltration heating method can be divided into three methods: saltwater frying, saltwater boiling and saltwater steaming. The methods commonly used in modern times are saltwater frying and saltwater steaming methods (Li et al., 2018; Si and Zhao, 2019; Zhou et al., 2021).

At present, Chinese Pharmacopoeia records: salt should be added to the appropriate amount of water dissolved, filtered, standby. Unless otherwise specified, 2 kg of salt should be used for every 100 kg of processed products. In addition, there is salt steaming method, pharmacopoeia provisions: general 100 kg processed products should add water or the provisions of the excipients 20–30 kg. There are more salt processing methods in the local processing standards than in the pharmacopoeia. For example, salt frying and salt steaming are recorded in the “*Processing Standards of TCM Medicinal Slices in Henan Province (河南省中药饮片炮制规范)*”. Shanghai province recorded salt-fried and saltwater-fried. Beijing, Anhui, Hunan and other provinces contain two kinds of salt processing method: one is to fry after saltwater stirring, the second is stir fry first and then add saltwater. Although there is no clear quality requirement for salt in local processing standards, some provinces have certain requirements for saltwater preparation. As shown in Table 1 (Wang et al., 2017). However, at present, the preparation process of saltwater is not clear, and there is no clear index for its quality control. Salt processing can affect the dissolution, efficacy and toxicity of relevant components of TCM. In order to ensure the quality of medicinal slices and the safety and effectiveness of clinical medication, the quality standard of saltwater should be further defined.

**TABLE 1:** Chemical composition changes of TCMs after salt processed.

TCM	Species	Medicinal parts	Processing method	Composition change	References
AR	*Anemarrhena asphodeloides* Bge.	Dried rhizome	Salt frying	Sarsasapogenin↑	Dang et al. (2009); Ji et al. (2017); Lei et al. (2015); Li et al. (2011b); Shao et al. (2020); Song et al. (2014); Song et al. (2015); Yan et al. (2019); Zheng et al. (2020)
				Timosaponin AI↑	
				Timosaponin AII↑	
				Timosaponin AIII↑	
				Timosaponin I↓↑	
				Timosaponin Ia↑	
				Timosaponin BII↓↑	
				Timosaponin BIII↑	
				Timosaponin E↓	
				Timosaponin E1↓	
				Mangiferin↑	
				Neomangiferin↓	
				Isomangiferin↓	
				Vitexin↓	
				Polysaccharide↑	
PF	*Dry bark* L.	Dry ripe fruit	Salt frying	Psoralen↑	Hong et al. (2021); Li et al. (2021a); Li et al. (2017a); Li et al. (2021c); LV et al. (2020); Tao et al. (2016); Wang et al. (2020)
				Isopsoralen↑	
				Psoralidin↑	
				Bavachinin↓	
				Isobavaenin↓	
				Corylifolin↓	
				Isobavachalcone↓	
				Corylin↑	
				Neobavaisoflavone↓	
				Bakuchiol↓	
				4′-O-Methylbroussochalcone↑	
				Psoralenoside↓	
				Isopsoralenoside↓	
				Liposoluble components↓	
				Volatile components↓	
EC	*Eucommia ulmoides* Oliv.	Dry bark	Salt frying	Geniposidic acid↓	Deng et al. (2015) He et al. (2021); Zhu et al. (2019)
				Caffeic acid↓	
				Geniposidic↓	
				Pinoresinol diglucoside↓	
				Genipin↓	
				Total amino acids↑	
				Total flavonoids↓	
				Total polysaccharides↑	
				Chlorogenic acid↓	
				Mediresinol di-O-β-D-glucopyranoside↓	
				Syringaresinol-di-O-Glucoside↓	
				Medioresinol↑	
				Pinoresinol↑	
				Epipinoresinol↑	
				Ferulaldehyde↑	
ALR	*Alisma orientale* (Sam.) Juzep	Dry tuber	Salt water moistening	Alismol G↓	Cai and Lin. (2021); Cao et al. (2016); Xiang et al. (2021)
			Salt frying	Alismol A↓	
				Alismol↑	
				24- Acetylalisol A↑	
				Alismol B ↑	
				23-Acetylalisol B↑	
AOF	*Alpinia oxyphylla* Mip.	Dry ripe fruit	Salt frying	Chrysin↑	Su et al. (2016)
LLF	*Ligustrum lucidum* Ait.	Dry ripe fruit	Salt steaming	Specnuezhenide↓	Xiao et al. (2019); Zhang et al. (2021a)
			Salt frying	Oleanolic acid↓	
				Ursolic acid↓	
				Salidroside↑	
				Tyrosol↑	
				Methyl ursolate↑	
				Apigenin↓	
				Luteolin-7-O-β-D-glucoside↓	
				6′-O-Cinnamyl-8-epigenic acid↓	
				Oleuropein↓	
				Ligustaloside A↓	
				Oleuropeinic acid↓	
				Ligustrosidic acid↓	
PCC	*Phellodendron chinense* Schneid.	Dry bark	Salt frying	Total sugar↑	Li et al. (2021b); Xu et al. (2017); Zhang et al. (2017b)
				Protein↓	
				Uronic acid↑	
				Berberine↓	
				Palmatine↓	
				Jatrorrhizine↓	
				Phellodendrine↓	
				Magnoflorine↓	
				Obaculactone↓	
				Obakunone↑	
				Berberrubine↑	
ABR	*Achyranthes bidentata* B1	Dry root	Fry with salt particle	β -Ecdysterone↑	Zhang et al. (2017d)
			Salt frying	25 R-Inokosterone↑	
	.			25 S-Inokosterone↑	
PF	*Phyllanthus emblica* L.	Dry ripe fruit	Salt frying	Gallic acid↑	Li et al. (2011a); Li et al. (2019)
				Ellagic acid↑	
				Epicatechin↓	
MOR	*Morirtda officinalis* How	Dry root	Salt frying Salt steaming	Sucrose↑↓	Ding et al. (2021); Liu et al. (2016a)
				Kestose↑	
				Asperulosidic acid↑	
				Fructose↑	
				Nystose↓	
				1 F-Fructofuranosylnystose↓	
PS	*Plantago asiatica* L. or *Plantago depressa* Willd.	Dry ripe seed	Salt frying	Geniposidic acid↑	Gu et al. (2016); Guan et al. (2017)
				Acteoside↑	
				Isoacteoside↑	
				Polysaccharide↓	
CF	*Chaenomeles speciosa* (Sweet) Nakai	Dry nearly ripe fruit	Salt frying	Oleanolic acid↑	Kuang et al. (2019); Wei and He. (2020)
				Ursolic acid↑	
				Chlorogenic acid↓	
				Gallic acid↓	
				Total saponins↓	
				Reducing sugar↑	
CR	*Cuscuta australis* R. Br. Or *Cuscuta chinensis* Lam	Dry ripe seed	Baking after salt water moistening	Hyperoside↓	Chen. (2017); Liu et al. (2016b)
				Rutin↓	
	.			Quercetin↑	
				Kaempferol↑	
				Fatty oil↑	
AVLF	*Amomum villosum* Lour. *Or Amomum villosum Lour.var.xanthioides* T.L. Wu et Senjen *or Amomum longiligulare* T.L.Wu	Dry ripe fruit	Salt frying	Isoquercitrin↓	Zheng and Wang. (2021); Zheng et al. (2021)
				Quercitrin↓	
				Camphor↓	
				Borneol↓	
				Bornyl acetate↓	
SR	*Foeniculum vulgare* Mill	Dry ripe fruit	Salt frying	Baicalin↓	Wei et al. (2021)
				Flavone↓	
	.			Polysaccharose↑	
CRS	*Citrus reticulata* Blanco	Dry ripe seed	Salt frying	Limonin↓↑	Chen et al. (2021)
			Baking after salt water moistening and heatng	Nominic↓↑	
				Eriocitrin↑	
				Obakunone↑	
Compound	Constitute	Salt processed components	Processing method	Composition change	References
Psoraleae Fructus pill	PF	Salt processed PF	Salt frying	Rutin↑	Wu et al. (2021a)
	SR	Salt processed SR		Isobavaenin↑	
				Neobavaisoflavone↑	
				Corylifolin↑	
				Corylin↑	
				Isobavachalcone↑	
				Bavachinin↑	
				Anisaldehyde↓	
				Trans-anethole↓	
				Bakuchiol↓	
				Psoralen↓	
				Isopsoralen↓	
Wuzi Yanzong pill	Fried CR	Salt processed CR	Salt frying	Chlorogenic acid↑	Arab et al. (2019); Liao et al. (2021a)
	Lycii Fructus (LF, the dried ripe seed of *Lycium barbarum* L.)	LF	Salt steaming	Ellagic acid↑	
	Salt processed PS	Salt processed PS		Hyperoside↑	
	Rubi Fructus (RF, the dried fruit of *Rubus chingii* Hu)	Salt processed RF		Isoquercitrin↑	
	Steamed Schisandrae Chinensis Fructus (SCF, the dried fruit of *Schisandra chinensis*(Turcz.)Baill.)	Salt processed SCF		Acteoside↑	
				Astragalin↓	
				Kaempferol↓	
				Schisandrin↓	

The processing of TCM is gradually developed and enriched in the past dynasties. Its content is rich, method is diverse, and the processing methods are different in different places. As we all know, proper processing is directly related to the efficacy of clinical drugs. The degree of processing is not enough, the medicine can’t exert its efficacy, while the over processing, the efficacy of the medicine will be lost. Attention should be paid to the temperature and operation standard during processing. For example, the Ziziphi Spinosae Semen (the dried ripe seed of Ziziphus jujuba Mill. Var. *spinosa* (Bunge) Hu ex H. F. Chou) is slightly fried or fried yellow, its effective components will increase, and if it is stir-bake to brown, the content of its effective components will decrease, resulting in a decrease in medicine efficacy. Salt processing TCMs should be constantly innovated scientifically based on the different functions in clinical practice, so as to improve the quality of TCM, and make the processed products of TCM more standardized.

## Salt processing TCMS

Historically, salt processing products were abundant. The development of salt processing products is similar to salt processing method, and the Song Dynasty to the Ming Dynasty is the prosperous period of salt processing development, and a large number of salt processing products appeared, especially in the *Compendium of Materia Medica*, with more than 80 kinds of salt processing TCMs added. People’s understanding of TCMs gradually improved. (Si and Zhao, 2019). As shown in Supplementary Material S1.

## Salt processing purpose

It is recorded in “*Supplementary Records of Famous Physicians (名医别录)*”: salt is classed as medium grade, cold in nature and salty in taste. It has the functions of removing heat and cooling blood, softening hardness and dissipating stagnation, moistening dryness and relieving constipation, strengthening muscles and bones, introducing medicine into kidney channel, antisepsis and modifying the taste. It can be preliminarily seen that the function of salt processing mainly includes the following two aspects:

(1) Using the therapeutic effect of salt and combining with TCM to improve the curative effect: Ⅰ. Introducing medicine into the kidney channel to treat disease, such as Citri Reticulatae Pericarpium (the dried ripe pericarp of *Citrus reticulata* Blanco), Aconiti Lateralis Radix Praeparata (the processed product of the child root of *Aconitum carmichaelii* Debx.), Coicis Semen (the dried and ripe kernel of *Coix lacrymajobi* L.var.*mayuen* (Roman.) Stapf) and Anemarrhenae Rhizoma (the dried rhizome of *Anemarrhena asphodeloides* Bge.). Ⅱ. Enhance the curative effect of treating hernia, relieving pain and reducing urine, such as Gendarussae Herba (the dried ripe fruit of *Foeniculum vulgare* Mill.), Euodiae Fructus (the dried near ripe fruit of *Euodia rutaecarpa* (Juss.) Benth., *Euodia rutaecarpa* (Juss.) Benth. Var. *Officinalis* (Dode) Huang or *Euodia rutaecarpa* (Juss.) Benth. Var. *Bodinieri* (Dode) Huang) and Alpiniae Oxyphyllae Fructus (the dried ripe fruit of *Alpinia oxyphylla* Miq.). Ⅲ. Enhance the function of tonifying the kidney, such as Eucommia Cortex (the dried bark of Eucommia ulmoides Oliv.), Cuscutae Semen (the dried ripe seed of *Cuscuta australis* R.Br. Or *Cuscuta chinensis* Lam.) and Psoraleae Fructus (the dried ripe seed of *Psoralea corylifolia* L.). Ⅳ. Enhance the functions of nourishing Yin, falling fire and moistening dryness, such as Anemarrhenae Rhizoma, Phellodendri Chinensis Cortex (the dried bark of *Phellodendron chinense* Schneid.) and Alismatis Rhizoma (the dried tuber of *Alisma orientale* (Sam.) Juzep.). Ⅴ. Alleviate the pungent and dryness toxicity of TCMs, such as Psoraleae Fructus and Alpiniae Oxyphyllae Fructus.
(2) Using the preservative, hypertonic, electrolyte balance and other chemical properties of salt to processed TCMs, so as to make them convenient for application. Ⅰ. Modify the taste and odor, reduce or eliminate the toxicity or side effects of TCMs, such as Euodiae Fructus and Ricini Semen. Ⅱ. Minerals and shell TCMs are hard and difficult to crush. The purpose of salt processing is to facilitate crushing, such as muscovite. Ⅲ. Using the preservative properties of salt to make medicines easier to preserve, such as Aconiti Lateralis Radix Praeparata, Citri Reticulatae Pericarpium, Toosendan Fructus (the dried ripe fruit of *Melia toosendan* Sieb.et Zucc.), *etc.* (Liu, 2003; Xu, 2005; Qian et al., 2018)

In short, salt processing is to use the therapeutic effect of salt to cooperate with medicine to improve the curative effect or use its physicochemical properties to processed medicine to make it convenient for application, generally the former. According to the clinical needs, TCMs in the processing of salt, or take its salty taste, or take its cold nature to facilitate treatment.

## Effect of salt processing on chemical composition

The research of TCM processing mechanism is to explore the principle of TCM processing to increase efficacy, reduce toxicity, moderate medicinal or produce new efficacy. At present, the mechanism of salt processing is mainly studied by chemical and pharmacological methods to study the influence of salt processing on the material basis and pharmacodynamic effect of TCMs. This research model has achieved a series of achievements (Cai et al., 2012; Tao et al., 2018; Tao et al., 2019a), which has played a certain role in promoting the development of TCM processing research.

In the process of processing, the chemical composition of TCM has undergone complex changes (Table 1), so as to play the role of processing to reduce toxicity and increase efficiency. Process of salt processing influence on TCM can be divided into the influence of the salt and heating. Salt is one of the commonly used excipients for the processing of TCM. It mainly contains NaCl, MgCl_2_, CaSO_4_, Zn, Ba, etc., which may affect some components of TCM, and the influence caused by the heating temperature that may destroy or decompose some components during salt processing. In this study, the effect of salt processing on chemical composition of TCM in recent 5 years were summarized.

### Anemarrhenae Rhizoma (AR)

Shao et al. used high performance liquid chromatography-evaporative light scattering detector (HPLC-ELSD) to determine the content of saponins in raw and salt processed AR. The results showed that the content of timosaponin BII decreased and the content of timosaponin AIII increased after salt processed. It is speculated that part of timosaponin BII may be transformed into timosaponin AIII (Shao et al., 2020). Zheng et al. determined the content of timosaponin AI, AII, AIII, BII, BI, I and Ia by ultra-performance liquid chromatography tandem mass spectrometry (UPLC-MS/MS). The results showed that the contents of seven timosaponin were all increased after salt processed (Zheng et al., 2020). Ji et al. established HPLC-MS/MS method to analyze the contents of timosaponin E1, BII, BIII, I, AII and AIII of raw and salt processed AR. The results showed that the contents of timosaponin AII and AIII were not significantly changed, the contents of timosaponin E1, BII and I were decreased, while the contents of timosaponin BIII were increased after salt processing. It is speculated that the timosaponin E1, BII and I may be transformed into timosaponin BIII during processing (Ji et al., 2017). Yan et al. used HPLC-charged aerosol detector (CAD) to compare the content changes of timosaponin AIII, BII, BIII and E in raw and salt processed AR. The results showed that the content of timosaponin AIII and BIII increased while the content of timosaponin E decreased after salt processing (Yan et al., 2019). Dang et al. also used HPLC-ELSD method to analyze the content changes of sarsasapogenin in raw and salt processed AR. The results showed that the content of sarsasapogenin in salt processed AR was slightly higher than that in the raw. It was speculated that part of the saponins decomposed during heating with salt, which made the content of sarsasapogenin was higher after salt processed (Dang et al., 2009).

Shao et al. used HPLC-ultraviolet detector (HPLC-UV) to determine flavonoids in AR. The results showed that the content of neomangiferin and mangiferin decreased after salt processed, while the content of isomangiferin did not change significantly (Shao et al., 2020). Li et al. compared the content changes of mangiferin in different processed products of AR, and found that the content of mangiferin in salt processed AR was the highest. On the one hand, some glycosidic bonds in neomangiferin were broken by heating, and on the other hand, the structural changes of isomangiferin caused by the addition of salt (Li X. et al., 2011). Song et al. used HPLC method to determine the content changes of vitexin, mangiferin, neomangiferin and isomangiferin in raw and salt processed AR. It was found that the content of vitexin decreased after salt processed. The content of neomangiferin and isomangiferin decreased, while mangiferin increased after salt processed (Song et al., 2014, 2015).

Lei et al. used UPLC-MS/MS method to compare the chemical composition of AR before and after processing, and the results showed that the polysaccharide content in salt processed AR was higher than that of raw AR (Lei et al., 2015). Therefore, it was speculated that the increase of polysaccharide content in AR processed with salt might be caused by the rupture of glycopeptide bonds of some glycoproteins during processing.

### Psoraleae fructus (PF)

Li et al. used UPLC method to simultaneously determine the content of 10 chemical components, including psoralen, isopsoralen, psoralidin, bavachinin, isobavaenin, corylifolin, isobavachalcone, corylin, neobavaisoflavone, and bakuchiol in raw and salt processed PF. It was found that with the extension of stir-frying time, the coumarin components decreased first, then increased and then decreased, while the total contents of flavonoids and 10 components decreased. The possible reason is that heating promotes the mutual transformation of coumarin glycosides and coumarins in PF (Li K. et al., 2017). Tao et al. found that the content of 4′-O-methylbroussochalcone increased significantly, while the content of bavachinin decreased significantly in salt processed PF, which may be caused by the degradation and transformation of bavachinin under high temperature (Tao et al., 2016). Li et al. analyzed potential hepatotoxic components in PF by HPLC, and found that the contents of psoralenoside, isopsoralenoside and bakuchiol decreased in the salt processed PF by stir frying after moistening with saltwater, which may be related to water processing promoting the degradation of glycosides and heating accelerating the decomposition of bakuchiol (Li Z. et al., 2021). Hong et al. found that compared with raw PF, the content of psoralen and isopsoralen increased slightly in salt processed PF, which may be transformed from psoralenoside and isopsoralenoside in the processing process (LV et al., 2020). This shows that the salt processed PF can enhance the effect of warming kidney and helping Yang, tonifying kidney and regulating Qi (Wang et al., 2020), and the contents of toxic components such as neobavaisoflavone, bavachin and isobavachalcone were greatly reduced, which may be caused by the instability degradation of A ring of dihydroflavonoids under high temperature. In addition, bakuchiol is easy to be oxidized and volatilized during processing heating, which is the main reason for the decrease of its content (Hong et al., 2021). These studies indicate that salt processing can ensure the effectiveness and safety of PF.

Li et al. investigated the effects of salt processed on the liposoluble and volatile components of PF by GC-MS, and found that salt processing could reduce the extraction rate of liposoluble components, increase the types of liposoluble components, decrease the relative content of monoterpenoids, increase the relative content of sesquiterpenoids, and increase the types of volatile components (Li H. et al., 2021). Liposoluble and volatile components in PF are not only its active components, but also the material basis of dry-toxicity. Salt processing can reduce the total content of liposoluble and volatile components, adjust the relative content and types of components, and produce new components, which may be one of the mechanisms of enhancing effect and reducing toxicity of salt processed PF.

### Eucommiae cortex (EC)

Zhu et al. established the fingerprint of raw and salt processed EC, and found that the response of geniposidic acid, caffeic acid, geniposidic, pinoresinol diglucoside and genipin showed a decreasing trend after salt processing (Zhu et al., 2019). Although the active components in EC were decreased after processing, the plasma concentration of salt processed EC was increased (He et al., 2021). Deng et al. determined the contents of total amino acids, total flavonoids, total polysaccharides, geniposidic acid, chlorogenic acid and pinoresinol diglucoside in raw and salt processed EC by HPLC-UV method. It was found that the contents of other components decreased except the total contents of amino acids and polysaccharides (Deng et al., 2015). Liu et al. found that the contents of geniposidic acid, chlorogenic acid, pinoresinol diglucoside, (+)-mediresinol di-O-β-D-glucopyranoside, syringaresinol-di-O-glucoside decreased, while the contents of (−)-medioresinol, pinoresinol, epipinoresinol, ferulaldehyde increased in salt processed EC (Liu et al., 2011). Therefore, it is speculated that the stability of some macromolecular components in EC is poor, and some chemical bonds are easy to break when heated, resulting in the formation of small molecules. Moreover, the addition of salt will affect the ionic strength of the reaction system, thus promoting or inhibiting the mutual transformation of some active components in EC. In addition, under high temperature conditions, flavonoids will undergo hydrolysis, oxidation and dissociation reactions, resulting in a decrease in the content of its chemical components. The glycosidic bond of lignan glycosides was broken, and the aglycones of lignan glycosides were increased significantly after salt processing, and the contents of iridoids were decreased due to oxidation or polymerization of iridoids under heating conditions. The content of chlorogenic acid was decreased after salt processing, and ferulaldehyde increased, which may be due to the decomposition of chlorogenic acid to caffeic acid and quinic acid under heating condition, and the dehydration reaction of caffeic acid to ferulaldehyde. In addition, the rubber silk in raw EC makes active components difficult to dissolve, and salt can affect the heat and mass transfer performance of cells, so that EC is more vulnerable to damage during processing, and the active components are more easily dissolved, thus improving the extraction rate of amino acids and polysaccharides. It also provided scientific basis for the traditional processing standard of “stir-fried silk” of EC (Yang et al., 2007; Zhang et al., 2009; Liu et al., 2011).

### Alismatis rhizoma (ALR)

Xiang et al. used UPLC-quadrupole time-of-flight tandem mass spectrometry (UPLC-Q-QOF-MS) to identify the chemical components in raw and salt processed ALR, and found that the contents of alismol G and alismol A decreased significantly after salt processing (Xiang et al., 2021). Cao et al. used HPLC to study the effects of salt processing on the contents of alismol, 24- acetylalisol A, alismol B and 23-acetylalisol B in ALR. Compared with raw ALR, alismol, 24- acetylalisol A, alismol B in the processed products increased, thus enhancing the diuretic effect of the processed products (Cao et al., 2016; Cai and Lin, 2021).

Wen et al. established the fingerprint of raw and salt processed ALR by HPLC. The raw and salt processed ALR had 14, 15 common peaks respectively, and their peak areas were different to varying degrees. Peak two was not found in the processed products, while peak eight and peak 16 appeared in the processed products, indicating that salt processing could affect the composition and content of chemical components of ALR (Wen et al., 2018). Han et al. used nuclear magnetic resonance technology to study the chemical composition differences between raw and salt processed ALR. Compared with raw ALR, the salt processed ALR has obvious changes in the high field area, indicating that salt processing has a greater influence on the chemical composition of ALR. It is suggested that dehydration, deacetylation and oxidation of the alismatol may occur during the process of salt processed ALR (Han et al., 2016).

### Alpiniae oxyphyllae fructus (AOF)

Su et al. proved that the content of chrysin increased after processing with salt (Su et al., 2016). Huang et al. used gas chromatography-MS (GC-MS) to study the volatile oil components in raw and salt processed AOF. The results showed that 33 compounds existed in raw and salt processed AOF, 35 compounds disappeared while 16 compounds increased after salt processing (Huang et al., 2008). Li et al. compared the chemical composition of volatile oil in raw and salt processed AOF, 77 peaks were detected in raw AOF, 94 peaks were detected in salt processed AOF, and 36 common peaks were detected both in raw and salt processed AOF (Li et al., 2015). The composition and relative content of volatile oil before and after processing of AOF changed greatly.

Sun et al. found that eudesm-11-en-1α-ol, 9,10-dehydroiso-longifolene, gurjunenepoxide, 1R, 4R, 7R, 11R-1, 3, 4, 7-tetramethyltricyclo [5.3.1.0 (4,11)]-undec-2-ene only existed in salt processed AOF (Sun et al., 2015). Li et al. established the fingerprint of raw and salt processed AOF by HPLC, and the results showed that two new chromatographic peaks (one was 5-hydroxymethylfurfural) appeared in salt processed AOF, and the relative contents of seven characteristic peaks changed (Li et al., 2010). Although some scholars have studied the fingerprint of chemical components before and after salt processing in TCM, and confirmed that certain quantitative and qualitative changes occur to chemical components after salt processing, the specific effects of these changes on pharmacological effects and their influencing mechanisms remain to be further studied.

### Ligustri lucidi fructus (LLF, the dried ripe fruit of *Ligustrum lucidum* ait.)

Zhang et al. used LC-UV method for the first time to study the processing dynamics of two kinds of salt processed LLF by salt steaming and salt frying. In salt steaming LLF, the contents of oleanolic acid and ursolic acid decreased slightly (about 4%) and specnuezhenide decreased significantly (10%–20%), while the contents of salidroside (70%–120%) and tyrosol (180%–240%) increased significantly. With the extension of frying time, the content of oleanolic acid and ursolic acid in salt frying LLF rise first and then gently decreased, and at the end of processing, it was at the same level with the raw LLF. The contents of salidroside and tyrosol increased, while specnuezhenide decreased significantly. The dynamic changes of components in salt steaming and salt frying of LLF were basically the same (Zhang H. et al., 2021). Xiao used UPLC-Q-TOF-MS to analyze the chemical composition in raw and salt processed LLF. After salt processing, the peak intensity of salidroside and methyl ursolate were increased and the peak intensity of apigenin, luteolin-7-O-β-D-glucoside, 6′-O-cinnamyl-8-epigenic acid, oleuropein, ligustaloside A, oleuropeinic acid and ligustrosidic acid decreased. In addition, the variety of compounds of LLF increased after salt processing, which shows that the chemical components of LLF have changed significantly before and after salt processing (Xiao et al., 2019).

### Phellodendri chinensis cortex (PCC)

Zhang et al. determined the contents of total sugar, protein and uronic acid of PCC before and after salt processing by phenol-sulfuric acid method, Coomassie bright blue method and sulfate-carbazole method. It was found that the contents of total sugar and uronic acid increased, while the contents of protein decreased slightly after salt processing, which might be due to the chemical transformation of polysaccharides during high temperature during salt processing. And the immune function of polysaccharides from PCC was more significant after salt processing, indicating that salt processing plays an important role in immunosuppression (Zhang et al., 2017b). Li et al. used UPLC-MS/MS method to determine the content of 10 components in raw and processed PCC, in order to find the quantitative change rule of active components in PCC at different frying temperatures and frying times. It was found that the content of berberine, palmatine, jatrorrhizine, phellodendrine, magnoflorine and obaculactone decreased during the salt processing process. The content of obakunone was higher when it was fried at 170°C–180°C for 4 min. The contents of berberrubine showed an upward trend, and the changes of the 10 components were the most obvious when frying at 180°C. This may be related to the conversion of berberine, palmatine and jatrorrhizine into them secondary metabolites with the increase of processing temperature and time (Xu et al., 2017; Li L. et al., 2021).

#### Achyranthis bidentatae radix (ABR, the dried root of *Achyranthes bidentata* Bl.)

Zhang et al. compared the effects of two salt processing methods on the content of *ß*-ecdysterone, 25 R-inokosterone and 25 S-inokosterone in ABR. It was found that the content of three compounds in ABR after salt processing was higher than that in raw, and the main factors affecting the content of compound are frying temperature and time, while the salt content and type are not very important. In the process of stir-frying ABR with salt water, after the ABR is moist with salt water, part of the water-soluble components dissolve out and cause the bottom of the pot to be wet and the temperature is not easy to control, too low temperature can not reach the effect of salt frying, too high is easy to stir-bake to brown. The best frying time of salt processed ABR prepared with salt water is 15 min, while the best frying time of salt processed ABR mixed with salt particle is 4 min. This may be related to the fact that the experimental operating conditions of salt particle stir fry are easier to control than salt water stir fry (Zhang Z. et al., 2017).

Meng et al. used GC-MS technology to analyze and compare volatile components in raw and salt processed ABR. Compared with raw ABR, 10 compounds were added and 17 compounds were disappeared in the salt processed ABR (Meng et al., 2017).

### Phyllanthi fructus (PF, the dried ripe fruit of *Phyllanthus emblica* L.)

Li et al. used HPLC method to determine the contents of gallic acid, ellagic acid and epicatechin in raw and processed PF to explore the effect of salt processing on its main chemical components. It was found that the content of gallic acid and ellagic acid increased and the content of epicatechin decreased after salt processing compared with the raw product (Li et al., 2019). There are complex polyphenol compounds in PF, and the basic composition unit is gallic acid. Ellagic acid is a dimeric derivative of gallic acid and has the chemical structure of polyphenol dilactone. With the increase of processing temperature, the tissue structure of medicinal materials became loose, and the rupture of plant cells increased the dissolution of chemical components, and the glucoside compounds containing gallic acid group decompose at high temperature, producing gallic acid and the corresponding aglycone and other compounds, resulting in the increase of the content of gallic acid. Under the action of polyphenol oxidase, the related chemical bonds of some polyphenolic compounds were broken and transformed into ellagic acid, resulting in the increase of the content of ellagic acid. Epicatechin is a compound with 2-phenyl benzopyran structure. Due to the five hydroxyl groups in the molecular structure, it has strong reducibility. With the increase of processing temperature, it is easy to occur oxidation reaction, resulting in a significant decrease in the content of epicatechin in salt processed PF (Li L. et al., 2011).

### Morindae officinalis radix (MOR, the dried root of *morinda officinalis* how)

Ding et al. analyzed the oligosaccharides and iridoids of MOR after salt processing, and found that the peak area of sucrose, kestose and asperulosidic acid increased significantly, may be because the texture of the medicinal materials changed after salt processing, the structure became more loose, and some of the active ingredients were easy to be decocted, which indicated that salt processing had a certain scientific basis (Ding et al., 2021). Liu et al. found that fructose content increased (*p* < 0.05), sucrose, nesose and 1 F-fructofuranosylnystose decreased after salt steamed MOR. It was preliminarily speculated that in the process of heating and steaming, the carbohydrate components of MOR were transformed into fructose and other components, which played a certain role in alleviating the dryness nature of MOR, and provided basis for further study on the salt processing mechanism of MOR (Liu F. et al., 2016).

### Plantaginis semen (PS, the dried ripe fruit of *Plantago asiatica* L. Or *Plantago depressa* willd.)

Guan et al. found that the content of geniposidic acid, acteoside and isoacteoside in processed PS was higher than that in raw products, while the content of polysaccharide in raw products was higher than that in processed products (Guan et al., 2017). Geniposidic acid belongs to the iridoids component, while acteoside and isoacteoside belong to phenylethanoid glycosides component. Modern pharmacological studies show that the iridoids and their glycosides have strong antioxidant activity, among which geniposidic acid has the strongest antioxidant activity (Gu et al., 2016). Therefore, this may be the pharmacodynamic substance basis of the synergistic effect after salt processing of PS.

### Chaenomelis fructus (CF)

Wei et al. concluded that the content of total organic acids decreased, the content of oleanolic acid and ursolic acid increased, the content of chlorogenic acid and gallic acid decreased, and the content of total saponins decreased after salt processed. The composition may be damaged due to the longer heating time with salt (Wei and He, 2020). The contents of total sugar and reducing sugar in different processed CF were determined by Kuang et al. Because the hard texture of CF affected the dissolution of components, it was found that the texture of CF was relatively loose after salt processing, which was conducive to the dissolution of polysaccharides in water, so the content of polysaccharides increased after heating and frying (Kuang et al., 2019).

### Cichorii radix (CR)

Liu et al. determined the contents of hyperoside, rutin, quercetin and kaempferol in salt processed CR by HPLC, and found that flavonoid glycosides including hyperoside and rutin decreased, while the flavonoid aglycones including quercetinand kaempferol increased. The possible reason is that the processing of CR has a heating process, hyperoside and rutin are glycosides, heating hydrolysis into aglycone and sugar, the aglycone of the two for quercetin and kaempferol. Therefore, the detection rate of flavonoids was increased and the content of total flavonoids was increased (Liu Y. et al., 2016). In addition, Chen et al. found that the content of fatty oil increased after salt processing (Chen, 2017).

### Amomum villosum lour fructus (AVLF, the dried ripe fruit of *Amomum villosum* lour., *Amomum villosum* lour.var.xanthioides T.L.Wu et senjen or *amomum longiligulare* T.L.Wu)

Zheng et al. used HPLC method to investigate the effects of salt processing on the contents of isoquercetin and quercetin in AVLF. The results showed that the contents of isoquercetin and quercetin decreased after processing, but the ratio of the two compounds remained stable at about 0.2060 (Zheng and Wang, 2021). Zheng et al. also used GC-MS method to determine the content of camphor, borneol and bornyl acetate. The results showed that the content of camphor, borneol and bornyl acetate all decreased after salt processing (Zheng et al., 2021).

### Other

Yu et al. identified 18 volatile components in raw FF and 16 volatile components in salt processed FF. The results showed that the relative content of trans-anethole decreased, while D-limonene increased after salt processed (Yu et al., 2016). Wei et al. determined the content changes of baicalin, total flavone and polysaccharide in Scutellariae Radix (SR, the dried root of *Scutellaria baicalensis* Georgi) before and after salt processing by HPLC, and found that the contents of baicalin and total flavone showed a decreasing trend while the content of polysaccharide increased after salt processing (Wei et al., 2021). Jiao et al. established HPLC-ELSD fingerprint before and after salt processing of Dipsaci Radix (DR, the dried root of *Dipsacus asper* Wall. Ex Henry), and found that the content of each component in the fingerprint varied in raw and processed DR, and there was no peak nine in salt processing products, indicating that the chemical composition and relative content of medicinal materials changed after processing (JIao and Wu, 2017). Chen et al. concluded that the contents of limonin and nosemilin decreased in salt frying Citri Reticulatae Semen (CRS, the dried ripe seed of *Citrus reticulata* Blanco) and the contents of eriocitrin, limonin, nosemilin and obakunone increased after baking after salt water moistening and heating (Chen et al., 2021).

### Compound medicines

Li et al. studied the fingerprint of Suoquan pill and found that the average relative area of 11 common peaks increased significantly after salt processed AOF. After processing AOF with salt, the effect of Suoquan pill on warming kidney and reducing urine was obviously enhanced, which may be related to the change of the content of the ingredients in AOF(Li M. et al., 2017).

Wu et al. studied the correlation between composition difference of Psoraleae Fructus pills before and after salt processing and antidiarrheal effect. It was found that the contents of flavonoids such as rutin, isobavaenin, neobavaisoflavone, corylifolin, corylin, isobavachalcone and bavachinin increased, while the contents of volatile oil such as anisaldehyde, trans-anethole and bakuchiol and coumarin components such as psoralen and isopsoralen decreased in salt processed Psoraleae Fructus pills, including salt processed PF and salt processed SR (Wu Q. et al., 2021).

Liao et al. determine the content of main chemical composition of Wuzi Yanzong pills after processed with salt by HPLC-diode array detector, the results showed that salt processed Wuzi Yanzong pills can promote the dissolution of chemical composition, and the content of chlorogenic acid, ellagic acid, hyperoside, isoquercitrin, acteoside increases. The results suggest that processing may enhance the effect by promoting effective constituent dissolution, and modern studies have shown that ellagic acid can reduce testicular injury and improve sperm quality (Arab et al., 2019). It is suggested that ellagic acid may play an important role in nourishing kidney and essence of Wuzi Yanzong Pill (Liao et al., 2021a).

Most scholars studied the effect of salt processing on chemical composition of TCM mainly through the changes of chemical composition of medicine before and after salt processing. Temperature is only one of the factors that affect the chemical composition of TCM during salt processing. In addition, due to the infiltration of salt water in the process of salt processing, the texture of Chinese medicinal materials will become more loose and easy to be crushed, increasing the surface area in contact with the solvent, so as to be more conducive to the dissolution and transfer of active ingredients. Furthermore, the role of supplementary salt in the process of salt processing can not be ignored and needs further study. For example, salt can destroy the colloidal substances formed by polysaccharide and mucinous substance in TMC such as EC and ALR, so as to avoid affecting the dissolution of chemical components in the process of processing, and NaCl in salt is also conducive to the dissolution of berberine, palmatine and jatrorrhizine in PCC(Zhang et al., 2012). In addition, the main component of salt is NaCl, which can adjust the osmotic pressure of human tissues, and can promote gastric juice secretion and protein absorption when entering into the stomach, and can enhance urinary function and diuretic effect (Luo et al., 2017).

## Effect of salt processing on pharmacoloy

Salt combined with TCMs can enhance the functions of tonifying liver and kidney, nourishing Yin and falling fire, curing hernia and relieving pain. Therefore, salt processing method is mostly used in TCM, which has the function of tonifying kidney and solidifying essence, treating hernia and relieving pain, diuresis and purging phase fire. In this study, the effect of salt processing on pharmacology of TCM in recent 5 years were summarized.

### Enhance the effect of tonifying kidney

The main function of salt processing for medicine is introduce drugs into the kidney channel, the combination of PF and FF after salt processing has more significant curative effect. Wu et al. discussed the effect of salt processing on the components and antidiarrhea efficacy of Psoraleae Fructus pills and found that salt processing on Psoraleae Fructus pills can promote the decoction of flavonoids, inhibit the decoction of volatile oil and coumarin, which may be one of the key factor of salt processing to enhance its antidiarrhea effect (Wu Q. et al., 2021) According to the TCM processing theory of “salt processing into the kidney meridian”, Suoquan pill composed by salt processed AOF can significantly reduce the content of blood urea nitrogen and creatinine in the serum of kidney-yang deficiency mice, and has a certain repair effect on the kidney injury of model mice. It is scientific and correct to use salt processed AOF instead of raw AOF in Suoquan pill (Shuai et al., 2018). Some studies have investigated the effect of salt processed Wuzi Yanzong pill on the apoptosis of antispermatogenic cells in rats, found that in the salt processed group, the levels of verbascoside, astragalin, kaempferol and schisandrin higher than the raw product group, and salt processed group of apoptosis of antispermatogenic cells effect significantly enhanced may be associated with the change of chemical composition content after processing (Liao et al., 2021b). Wu et al. study showed that the levels of Na^+^-K^+^-ATP and Ca^2+^-Mg^2+^-ATP and the activity and content of total cholesterol and glucose were all significantly increased, while the content of triglyceride was decreased, and the effect of warming kidney and helping Yang was enhanced in rat after oral salt processed MOR (Wu W. et al., 2021). This is consistent with the effect of salt processed MOR focusing on the kidney for strengthening the kidney and Yang.

Compared with raw EC, salt processed EC could increase the level of bone mineral density, mineral content, estradiol, bone growth factor (BMP-2, VEGF, TGF-*β*, IGF-1, OPG), thymus, testicular index, and decrease serum calcium, phosphorus, parathyroid hormone, tartrate-resistant acid phosphatase, osteocalcin, tumor necrosis factor *a* (TNF-α), interleukin-6, procollagen type Ⅰ N-terminal propeptide, *ß* isomer of C-terminal telopeptide of type Ⅰ collagen and other biochemical indexes, and had a good effect on the prevention and treatment of rat osteoporosis induced by retinoic acid (Xiong and Zhao, 2020a; Xiong and Zhao, 2020b; Xiong and Zhao, 2020c). Salt processed EC could promote the proliferation and differentiation of human osteoblasts better than raw materials. Weng et al. showed that iridoids from EC may be the effective components of their kidney-nourishing and bone-strengthening effects (Weng et al., 2015). In addition, Tao et al. used prednisone to induce zebrafish osteoporosis model, and found that compared with raw ABR, salt processed ABR could significantly increase the amount of bone mineralization in zebrafish osteoporosis model, and significantly up-regulated the expression levels of transforming growth factor-β, p-Smad2/3 and Smad four proteins, down-regulated the expression levels of Smad7 protein, and the therapeutic effect of salt processed ABR was better than raw ABR (Tao et al., 2020). Yang et al. found that PF did not significantly promote bone formation of osteoblast, however it could significantly promote bone formation after salt processed (Yang et al., 2018). In addition, Li et al. found that salt processed PF can enhance the effect of anti-osteoporosis, which is consistent with the traditional concept of TCM that “salt processing can enhance the effect of tonifying kidney and strengthening bones” (Li N. et al., 2017).

### Enhance the effect of treating hernia, relieving pain and reducing urine

Mo et al. studied the analgesic effect of raw and processed CRS. The results showed that compared with raw CRS, salt processed CRS could increase the pain threshold of mice induced by hot plate stimulation, and significantly reduce the number of writhing body caused by intraperitoneal injection of acetic acid, which was consistent with the traditional theory that salt could enhance the effect of treating hernia and relieving pain (Mo et al., 2007).

Zhang et al. compared the effects of raw and salt processed RF on serum cyclic adenosine monophosphate (cAMP) and cyclic guanosine monophosphate (cGMP) in adenine-induced kidney-yang deficiency polyuria rats and its improvement on renal pathological changes. It was found that the effect of salt processed RF on increasing cAMP and decreasing cGMP was significantly enhanced. At the same time, the number of renal corpuscles and the enlargement of renal tubule lumen were improved. It can be seen that salt processed RF can regulate cAMP and cGMP in kidney-yang deficiency polyuria model rats, and improve renal pathology, which is consistent with the enhanced effect of salt processed RF on tonifying kidney and strengthening astringency (Zhang X. et al., 2021).

Gong et al. isolated and identified a number of compounds from salt processed AOF, and verified that 7-epi-teucrenone B had significant effects on the average tension and inhibition rate of detrusor muscle contraction in guinea pigs through the pharmacological test. It was revealed that 7-epi-teucrenone B in salt processed AOF may be the pharmacodynamic substance basis of warming kidney and reducing urine (Gong et al., 2017). This is consistent with the traditional understanding that salt processing introduce AOF into the kidney channel and strengthen the effect of warming the kidney and reducing urine.

### Enhance the effect of nourishing Yin, falling fire and moistening dryness

Salt, salty in taste and cold in nature, belong to Yin, could enhance the effect of nourishing Yin and clearing heat of TCM. Fan et al. compared the effects of AR-PCC before and after salt processing on glucolipid metabolism in type 2 diabetic rats, and salt processed AR-PCC had the best comprehensive effect on hypoglycemic and lipid regulation after salt processing. It may be related to the increased content of timosaponin BII, mangiferin, berberine and palmatine after salt processing (Fan et al., 2021). Fan et al. also studied the nourishing Yin and clearing heat effect of AR-PCC before and after salt processing on rats with kidney-Yin deficiency caused by glucocorticoid, and found that the effect of AR-PCC was enhanced after salt processing, which may be related to the up-regulation of the viscera index of epinephros and thyroid, and regulation the of content of cAMP and cGMP in plasma, inhibition the Na^+^-K^+^-ATP activity of liver, kidney and small intestine (Fan et al., 2020b). However, its pharmacodynamic substance basis needs further exploration. In addition, salt processed PCC could further enhances its bitter taste and cold nature, and introduces drugs into the kidney channel to enhance the function of nourishing Yin and reducing fire.Modern pharmacological studies show that PCC especially berberine has the most obvious effect on intestinal tissue, which can promote ulcer healing, protect mucosal barrier, inhibit inflammatory response, maintain microbial balance and enhance intestinal tension and peristalsis amplitude (Zhang L. et al., 2018). Zhao et al. studied that salt processing can enhance the efficacy of PCC on ulcerative colitis, restore the length of colon in mice with ulcerative colitis, reverse the general damage of colon, and inhibit local oxidative stress response (Zhao et al., 2021).

### Reduce or eliminate the toxicity or side effects

Salt processing is one of the traditional processing methods in China. Its function is not only to introduce drug into kidney channel, nourishing liver and kidney, but also to reduce toxicity by removing dryness. In the Chinese Pharmacopoeia (2020 edition), salt processing is used as the processing method of PF. Some studies have also found that salt processing can alleviate the side effects of PF on liver and kidney function of kidney-Yang deficiency rats, and relieve the dryness of raw PF. The alleviating effect of salt processing on PF dryness may be related to the regulation of gene expression of aquaporin in oral salivary gland (Liang et al., 2017). Xia et al. found that PF could alleviate dryness after salt processing, and could reduce the contents of cAMP/cGMP, TNF-α and Na^+^-K^+^-ATP level in kidney-Yang deficiency model induced by hydrocortisone and spleen deficiency model induced by Rhei Radix et Rhizoma, thus reducing liver injury (Xia et al., 2016). Cai et al. compared the contents of psoralen and isopsoralen between raw and salt processed PF, and found that the contents of these two components in salt processed PF were lower than that of raw PF, and the acute toxicity of salt processed PF was lower in normal mice, suggesting that the toxicity of PF was positively correlated with the content of psoralen and isopsoralen (Cai et al., 2017). In addition, Chen et al. found that different processed products of Toosendan Fructus (TF) had toxic on LO2 cells *in vitro*, and salt processing could reduce the hepatotoxicity of TF. The mechanism of processing detoxification may be related to the reduction of mitochondrial dysfunction (Chen H. et al., 2018). Moreover, salt processing can effectively reduce the hepatotoxicity of Euodiae Fructus (EF) and reduce the liver injury caused by EF. The mechanism of its detoxification may be related to alleviating the oxidative damage of liver tissue, improving the energy metabolism of liver tissue, reducing the release of inflammatory factors and regulating the expressions of apoptosis-related proteins Bcl-2 and Bax (Zhang S. et al., 2017; Zhang S. et al., 2018; Liu et al., 2018).

## Effect of salt processing on *in vivo* behavior

At present, the explanation of TCM processing mechanism mainly focuses on the changes of chemical components and pharmacological effects, while the study on absorption, distribution and metabolism of TCM active components before and after processing is not thorough enough to reveal the integrity and complexity of efficacy changes before and after processing. With the introduction of new techniques and methods, it is possible to comprehensively elucidate the processing mechanism of TCM, and it has become a hot spot in the study of the processing mechanism of TCM. This study summarized and analyzed the methods of salt processing based on animal processes, providing reference for the research on the mechanism of TCM processing, and it can bridge the changes of chemical composition and pharmacological effect of TCM before and after processing (Pan et al., 2018).

Zhang et al. analyzed the effect of salt processing time on the components of EC, and found that the longer processing time, the more loss of active components, the content of pinoresinol diglucoside increased at 0–1 h, the content of geniposide, geniposidic acid and aucubin increased significantly at 1–2 h, then the content decreased sharply. The absorption of EC in rats was increased 2 h after processing, indicating that reasonable processing time could promote the absorption of main components in EC in rats and improve the bioavailability of pharmacodynamic components in EC (Zhang et al., 2016). Wu et al. investigated the effect of salt processing on the pharmacokinetics of nemangiferin in AR. The pharmacokinetic parameters of neomangiferin in raw and processed group rats were: AUC_(0-t)_ of (43.913 ± 0.636) and (71.565 ± 1.950) mg/L*h, respectively. AUC_(0-∞)_ of (44.127 ± 0.673) and (71.805-1.926) mg/L*h, respectively. C_*max*_ of (4.539 ± 0.048) and (5.805 ± 0.119) mg/L, respectively. The results showed that salt processed AR could promote the absorption of neomangiferin and increased the bioavailability. It is speculated that the factors such as Cl^−^ in NaCl and heating may promote the absorption of neomangiferin during salt processing (Wu Y. et al., 2016).

Tang et al. compared the distribution of mangiferin in raw and salt processed AR in rat tissues, and found that mangiferin in raw and salt processed AR groups were concentrated in lung and kidney tissues, which was consistent with the characteristics of AR channel tropism, and the distribution content of salt processed AR in lung and kidney tissue was slightly higher than that of raw group (Tang et al., 2021). Zhang et al. used UPLC-MS/MS method to determine the difference in the absorption of alkaloids in the kidney tissues of rats in raw and salt processed PCC, and found that in the same time after administration, the concentration of phellodendrinethe, magnoflorine, jatrorrhizine, palmatine and berberine in the salt processed PCC in the kidney tissues was higher than that in the raw PCC (Zhang et al., 2020). Fan et al. took the total content of psoralen and isopsoralen in PF as indicators, and found that salt as an excipient in both frying and microwave processing could promote the absorption of effective constituent into the kidney (137% and 176%, respectively) (Fan et al., 2019). The above studies show that the kidney absorption effect of salt processed AR, PCC and PF were better than that of raw products, verified the processing traditional theory of salt processing introduced medicines into the kidney channel to enhance tonifying kidney, strengthening bone and anti-osteoporosis effect.

Yan et al. investigated the effects of different processing methods on the intestinal absorption of anti-osteoporosis components in PF by everted gut sac method and *in vivo* intestinal absorption model. The results showed that, compared with wine immersion method, steaming method, thunder gong method and stir-frying method, salt processing can improve the intestinal absorption of psoralen, isopsoralen, neobavaisoflavone, bavachin, isobavachalcone, corylifol A and bakuchiol. This is consistent with the salt processed PF can enhance its effect on osteoporosis (Yan and Gao, 2020). Wu et al. used unidirectional intestinal perfusion model in rats, and found that the absorption rate parameters and effective permeability coefficient of the active component of nootkatone of AOF in small intestine increased after processed with salt. It was speculated that the absorption rate of processed with salt was better than that of raw one, which might be related to the effect of salt processing on enzyme activity, and this may be one of the mechanisms of enhancing antiuria after salt processing (Wu S. et al., 2016).

Based on 16 S rDNA sequencing technology, Fan et al. analyzed the effects of AR-PCC before and after salt procesing on intestinal microflora of type 2 diabetic rats, and found that compared with the model control group, raw and salt processed AR-PCC could significantly reduce blood glucose level (*p* < 0.01) and increase body weight (*p* < 0.05), improve the intestinal flora diversity, increase the beneficial bacteria of Class Clostrida, Romboutsia and *Bacteroides* abundance, reduce *Bacillus* acidi lactici, *bacillus* abundance, regulate and improve type 2 diabetes rat intestinal flora disorder, especially the group of salt processed AR-PCC had the best effect. These results suggest that the synergistic mechanism may be related to the regulation of intestinal microecology (Fan et al., 2020a).

Tao et al. found that salt processed ABR could improve the serum albumin level and the metabolism of glucose, lipids and amino acids in the tissues of osteoporosis model rats, thus preventing visceral diseases (Tao et al., 2019b).

The effect of salt processing on *in vivo* behavior of traditional Chinese medicine is mainly in absorption and metabolism. Because salt processing involves heating process, the change of temperature plays a crucial role in the structure of compounds, which leads to increased absorption of the drug as it enters the body. Serum pharmacochemistry could be used to directly isolate and identify the active material basis of TCMs from serum and study its pharmacokinetic characteristics. Metabolomics technology is characterized by high coverage of data acquisition and extensive network analysis by analyzing endogenous metabolites of organisms, it has been widely used by researchers of TCM (Jia et al., 2018; Miao et al., 2018), and the changes of endogenous and exogenous substances *in vivo* after oral salt processed TCM is worthy of further study by scholars and future scholars not only need to study the changes of chemical components *in vitro* during the processing process, but also need to clarify the changes of processes *in vivo*, and combine the two for comparative study, so as to better reveal the mechanism of salt processing of TCM.

## Salt processing technology

Box-behnken response surface method (Chen D. et al., 2018), variance analysis method (Zhang Y. et al., 2018), orthogonal design (Chong et al., 2021), central composite design (Lei et al., 2022) and other methods were adopted to improve and optimize the salt processing technology. In the optimization process, the factor of moistening time, frying temperature, frying time, salt dosage and the concentration of salt water on the dissolution rate of effective constituent were mainly investigated. With the development of the times, some scholars began to use microwave processing method and thermostatic drying method as new heating methods to replace the frying in the process of salt processing. For example, Chen et al. used orthogonal design method to explore the effects of microwave intensity, processing time and salt dosage on the processing technology of CR. It was found that the best microwave processing conditions of salt CR were as follows: salt content was 3%, moistening time was 2 h and then processed for 3 min in microwave over medium heat (Chen et al., 2017). Compared with the traditional processing heating method, this method has the advantages of simplicity, controllable conditions, fixed parameters of influencing factors, easy batch processing and saving labor and time.

## Deficiency of salt processing

In recent years, researchers have gradually deepened their research on salt processing TCM from simple research on the processing technology to the influence of salt processing TCM on chemical composition, pharmacological effect and *in vivo* behavior. However, some problems need to be paid attention to in the study of salt processing mechanism of TCM.

### Amount and type of salt

As for the amount of salt used in processing, no exact evidence was found by studying historical documents, and the current amount of salt is a summary of experience, lacking historical research and theoretical support, which needs further research and verification. As for the varieties of salt, *Compendium of Materia Medica* recorded salt, Qing salt, Bai salt. With the progress of science and technology, processing salt is gradually changed into salt. With the development of salt industry, there have been iodized salt and other salt added with a variety of trace elements on the market, and there is still a lack of basic research on the selection of salt types for processing. As for the amount and type of salt, a comparative study should be carried out on the type, source and purity of salt on the basis of systematic excavation and collation of ancient and modern literatures. In the future, I. Researchers can establish fingerprints to detect the differences between chemical components and trace elements in different types of salts; Ⅱ. To detect the changes of chemical composition of TCM before and after processing by using different kinds and doses of salt, and to analyze the role of salt, salt dosage and heating in the process; Ⅲ. Establish animal models to evaluate the efficacy of different types and doses of salt, and establish a standard specification for processing auxiliary salt.

### Salt processing method

The processing technology of TCM has been improved a lot after thousands of years of evolution. In the case of salt processing technology, from the original salt water boiling to later salt water immersion, salt water stir-fry, salt water steaming and other new methods continue to emerge. In addition, the current salt theory still generally follows the viewpoint of the Ming and Qing Dynasties, TCM was processed with salt by traditional method based on past experience, but in the actual operation due to the fire is difficult to control, resulting in poor repeatability and stability of salt processing quality. Fortunately, modern studies can use microwave processing method and thermostatic drying method, because medicinal slices being in a closed environment, the operation is convenient and quick, coupled with the controllability of time and firepower, so that the processed products have no focal spot, uniform color, fast drying speed, stable quality and better repeatability than the traditional method (Li and Huang, 2014; Zhang et al., 2017a). At the same time, future scholars can discuss the difference between microwave processing method or thermostatic drying method and frying method on the pharmacodynamics, and improve the salt processing method. In addition, the optimization of salt processing technology often takes the total content of one or several components as the index, if this component is the effective component of the medicinal materials remains to be discussed.

### Quality standard

TCM salt processing method has a long history, which is the accumulation and summary of clinical practice experience of doctors in past dynasties. In different periods, the varieties of TCM and excipient salt are different. Throughout history, however, salt processing method and processing excipient are lack of related standards, excipient content impact on the quality of TCM lack the evaluation standard. The quality of processed products is difficult to be guaranteed due to the lack of specific parameter standards, thereby cause the quality of TCM processed products on the market is uneven. With the development of modern science and technology, the emergence of colorimeter, the objective quantification of color of TCMs can be realized. The colorimeter can quantitatively and digitally determine the sensory characteristics of raw and processed TCMs, making the experience becomes an accurate, rapid popularization and strong operability technology. In order to better control the quality of salt processed TCMs, it is of great significance to establish the specific quality standard of the processed products. In addition, future scholars can also screen the differentially expressed components in raw and processing TCMs by serum pharmacochemistry combined with chemometrics, and use them as quality markers. This will help enterprises to standardize production, ensure the quality of TCM, maintain the development of TCM processed market.

### Processing mechanism

Many researchers are studying the processing mechanism of TCM. Some scholars analyze the influence of salt processing on TCM from the perspective of chemical composition and pharmacological action, but the principle of chemical composition increase or decrease after salt processing is not clear enough. Some scholars believe that chemical composition change after processing is the substance basis that leads to the change of efficacy, but whether it is an effective active ingredient needs to be verified by pharmacodynamics test. And in the future, Ⅰ. Scholars can screen the differentially expressed components in raw and processing TCMs by serum pharmacochemistry combined with chemometrics; Ⅱ. To study the mechanism of differentially expressed components by systems biology, Ⅲ. To verify the mechanism of TCMs processing in the level of cells, organoids and animals. In addition, some scholars study the chemical composition and pharmacodynamic changes of salt processed TCM with normal saline as the control, lack a comparative study of processed products and the same amount of salt group, it can not distinguish whether the resulting pharmacodynamic changes are salt processing or salt water. Some scholars compared the bioavailability of TCMs before and after salt processing from the pharmacokinetic perspective, but lack of exploration of exciphant salt on the endogenous substances interference absorption of active components. Future scholars should consider the influence of excipients on the activity and content of endogenous substances *in vivo* when doing pharmacokinetic studies. All in all, there is still a long way to go to explain the processing principle of salt processed, standardize, improve and innovate the processing technology, formulate the quality standard, and ensure the safety and effectiveness of clinical medication.

## Conclusion

The processing of TCM is an important part of the modernization of TCM. In modern times, salt is used as the processing salt of TCM. The commonly used salt processing methods include salt water washing, salt water infiltration, salt water infiltration heating and salt water stir-frying method. The salt processing of TCM is helpful to introduce drugs into the kidney channel and enhance the effect of nourishing Yin and falling fire. The pharmacological effect, chemical composition and *in vivo* behavior of TCM will change after salt processing.

Although at present, some drugs have made breakthrough progress in processing research. However, the systematic research on the salt preparation of traditional Chinese medicine is not perfect, which needs to be scientifically expounded by relevant scientific research institutions in the future, and can summarize the law of drug changes after treatment with different processing technologies, so as to maintain the advantages of traditional Chinese medicine in China, and study a more scientific traditional Chinese medicine processing theory on this basis, so as to better serve mankind. Although at present, some drugs have made breakthrough progress in processing research. However, the systematic research on the salt processing of TCM is not perfect, which needs to be scientifically expounded by relevant scientific research institutions in the future, and can also summarize the law of drug changes after treatment with different processing technologies, so as to maintain the advantages of TCM, and on this basis, a more scientific theory of TCM processing is studied so that Chinese medicine can better serve human beings.

## Glossary

TCM: Traditional Chinese medicine
AR: Anemarrhenae Rhizoma
HPLC: high performance liquid chromatography
ELSD: evaporative light scattering detector
UPLC: ultra-performance liquid chromatography
MS: mass spectrometry
CAD: charged aerosol detector
UV: ultraviolet
PF: Psoraleae Fructus
EC: Eucommiae Cortex
ALR: Alismatis Rhizoma
AOF: Alpiniae Oxyphyllae Fructus
GC: gas chromatography
LLF: Ligustri Lucidi Fructus
PCC: Phellodendri Chinensis Cortex
ABR: Achyranthis Bidentatae Radix
PF: Phyllanthi Fructus
MOR: Morindae Officinalis Radix
PS: Plantaginis Semen
CF: Chaenomelis Fructus
CR: Cichorii Radix
AVLF: Amomum Villosum Lour Fructus
FF: Foeniculi Fructus
SR: Scutellariae Radix
CRS: Citri Reticulatae Semen
LF: Lycii Fructus
RF: Rubi Fructus
SCF: Schisandrae Chinensis Fructus
TNF-α: Tumor necrosis factor α
cAMP: Cyclic adenosine monophosphate
cGMP: cyclic guanosine monophosphate
TF: Toosendan Fructus
EF: Euodiae Fructus.

## References

[B1] Arab H. H. Gad A. M. Fikry E. M. Eid A. H. (2019). Ellagic acid attenuates testicular disruption in rheumatoid arthritis via targeting inflammatory signals, oxidative perturbations and apoptosis. Life Sci. 239, 117012. 10.1016/j.lfs.2019.117012 31678279

[B2] Cai B. Qin K. Wu H. Cai H. Lu T. Zhang X. (2012). Chemical mechanism during Chinese medicine processing. Prog. Chem. 24 (4), 637–649.

[B3] Cai D. Lin Y. (2021). Effects of different processing methods on 23-O-acetylalisol B in rhizoma Alismatis from zichuan. Chin. J. Ethnomedicine Ethnopharmacy 30 (21), 45–48.

[B4] Cai T. Huang N. Wang L. Sun X. Li X. Sun R. (2017). Experimental comparison study on acute toxicity of different processing methods of Psoraleae Fructus on nomal mice. Chin. J. Pharmacovigil. 14 (12), 730–736.

[B5] Cao L. Li Q. Fang Q. Wang X. Zhou X. Yang Y. (2016). Effects of three processing methods on four triterpenoids in Alismatis Rhizoma. Chin. Tradit. Pat. Med. 38 (9), 1994–1998.

[B6] Chen D. Zheng K. Li W. Zhao Y. Zhang T. Li Y. (2018a). Optimization of preparing of allium tuberosum seed with salt water by box-behnken response surface method based on CRITIC weighted evaluation. J. Chin. Med. Mater. 41 (4), 859–862.

[B7] Chen H. TaN L. Huang Y. Yang K. Zeng C. (2018b). *In vitro* hepatotoxicity of different extracts of Melia chinaberry on human normal hepatocytes LO2. J. Chin. Med. Mater. 41 (8), 1869–1873.

[B8] Chen J. Chen B. Song Y. Yu H. (2017). Microwave processing technology of Salt Cuscuta seed was optimized by orthogonal design. Lishizhen Med. Materia Medica Res. 28 (12), 2929–2930.

[B9] Chen W. (2017). Determination of fat oil content and effect of processing method on its content in Dodder. J. Sichuan Traditional Chin. Med. 35 (2), 64–65.

[B10] Chen X. Wang L. Meng J. Zhang Y. Lin H. Cao H. (2021). Research progress of Citri Reticulatae semen. Chin. J. Exp. Traditional Med. Formulae 27 (6), 226–233.

[B11] Chong Z. Gao B. Luo C. Hu Y. (2021). Optimal processing technology for selection of salt-processed Cuscuta chinensis by orthogonal test. Shaanxi J. Agric. Sci. 67 (9), 69–72.

[B12] Dang X. Mao C. Lu T. Xu J. Wang J. (2009). Determination of mangiferin and sarsaparinogenin in Anemophyllum anemophyllum by HPLC. Chin. Traditional Herb. Drugs 40 (2), 236–238.

[B13] Deng C. Han L. Zhang Y. Jiang Y. (2015). Changes of chemical constituents in Eucommiae cortex before and after processed with salt. Chin. Tradit. Pat. Med. 37 (11), 2464–2468.

[B14] Ding Q. Liu X. Wei M. Huo W. Chen F. Wang L. (2021). Difference of fingerprints of oligosaccharides and iridoids in Morinda officinalis before and after stir-frying with salt-water. Mod. Chin. Medicine**23** (3), 512–517+547.

[B15] Fan B. Guo Y. Huang T. (2019). Evaluation of Psoralea coryfolia L processing technology based on tissue distribution characteristics. J. Liaoning Univ. Traditional Chin. Med. 21 (2), 56–58.

[B16] Fan S. Zhang C. Li X. Ao M. Yu L. (2020a). Effect of raw and salt processed herb pair anemarrhenae rhizoma-phellodendri chinensis cortex on gut microbiota of type 2 diabetic rats based on 16S T sequencing technique. Pharmacol. Clin. Chin. Materia Medica 36 (6), 150–156.

[B17] Fan S. Zhang C. Luo T. Wang J. Tang Y. Yu L. (2020b). Study on effect of nourishing Yin and clearing heat of zhimu-huangbo herb pair processed with salt on kidney-yin deficiency rats. Traditional Chin. Drug Res. Clin. Pharmacol. 31 (10), 1141–1146.

[B18] Fan S. Zhang C. Wang J. Li X. Luo T. Yu L. (2021). Effect of compatibility of anemarrhenae rhizoma-phellodendri chinensis cortex couplet medicines on glucolipid metabolism in type 2 diabetic rats before and after salt-processing. Chin. J. Exp. Traditional Med. Formulae 27 (3), 22–29.

[B19] Gong X. Li M. Wu S. Hu L. Hu C. (2017). Separation and pharmacodynamics of urine-reducing components in petroleum ether part of salt-processed Alpiniae oxyphyllae Fructus. Chin. J. Exp. Traditional Med. Formulae 23 (6), 13–18.

[B20] Gu C. ZengHui W. SiHao Z. Huang L. (2016). Analysis on chemical composiiitions of crude and salt-processed plantage asiatica L. By UPLC-Q-TOF/MS. Mod. Traditional Chin. Med. Materia Medica-World Sci. Technol. 18 (1), 77–81.

[B21] Guan S. Wang Y. Zhang Z. Li K. (2017). Contents determination for three active constituents of plantaginis semen before and after salt processing by HPLC wavelength switching method. Liaoning J. Traditional Chin. Med. 44 (8), 1711–1715.

[B22] Han W. Lin X. Guo N. Chen Q. Zhang T. Xu N. (2016). Changes of chemical constitution in Alismatis rhizoma after saline processing as revealed by 1 H NMR analysis. Chin. J. Magnetic Reson. 33 (1), 117–124.

[B23] He Y. Liu Y. Wang F. Zhang D. Yang M. (2021). Processing history and modern research progress of eucommia ulmoides. Mod. Chin. Med. 23 (4), 593–598.

[B24] Hong L. Wang Z. Tang X. Yuan H. (2021). Processing of Psoraleae Fructus based on combination of Leigong method and salt processed method and its hepatotoxicity evaluation. Chin. Traditional Herb. Drugs 52 (22), 6983–6989.

[B25] Huang Q. Hu C. Li X. Li X. Yang Z. Xie X. (2008). Study on difference between components in volatile oil of Fructus Alpinia oxyphylla before and after stir-fried with salt. China Pharm. 17 (5), 3–4.

[B26] Huang Q. Hu C. Yu L. Li X. Li X. Lin H. (2007). Study on the historical evolution of traditional Chinese medicine salt processing method. China Association of Chinese Medicine, 338–342.

[B27] Ji D. Su X. Huang Z. Zhang X. Lu T. (2017). Determination of eight constituents in Anemarrhenae Rhizoma before and after salt processing by HPLC-MS. Chin. Traditional Herb. Drugs 48 (9), 1784–1790.

[B28] Jia Z. Xu W. Ma K. Xiao H. (2018). Study on the quality and mechanism of traditional Chinese medicine based on metabolomics. Mod. Traditional Chin. Med. Materia Medica-World Sci. Technol. 20 (8), 1373–1380.

[B29] Jiao T. Wu C. (2017). HPLC-ELSD fingerprints Comparison of Rhizoma continuosa before and after processing. Lishizhen Med. Materia Medica Res. 28 (8), 1895–1896.

[B30] Kuang J. Chen B. Liang X. (2019). Determination of polysaccharide content in Chaenomelis Fructus with DNS method and content comparison of different processed products. J. North Pharm. 16 (7), 1–3.

[B31] Lei X. Chen X. Meng J. Sun Y. Wang S. (2022). Optimization of stir-frying with saltwater technology of Citrus reticulata by entropy weight method combined with central composite design-response surface method. China Pharm. 33 (2), 165–171.

[B32] Lei X. Zhang J. Li Y. Wang Q. Xue J. Su X. (2015). Exploring effective components of laxative effect of Anemarrhenae Rhizoma based on Chinese herbal processing theory. China J. Chin. Materia Medica 40 (7), 1283–1286. 26281547

[B33] Li H. Cao Y. Tian L. Shi Y. Zhang Z. Li K. (2021a). Five different processing methods induced variations in composition and content of liposoluble and volatile constituents of Psoralea corylifolia. Chin. Tradit. Pat. Med. 43 (9), 2418–2427.

[B34] Li H. Huang H. (2014). Comparing berberine content of Phellodendron amurense Rupr. with salt of two methods. Nei Mongol J. Traditional Chin. Med. 33 (13), 101–102.

[B35] Li K. Xu M. Zhou N. Zhang Z. (2017a). Determination of 10 components in stir-frying Psoralea Fructus with salt solution by different processing time. Chin. Traditional Herb. Drugs 48 (4).

[B36] Li K. Zhou N. Tian L. Zhang Z. (2018). Historical evolution of salt and salt processing of traditional Chinese medicine. J. Chin. Med. Mater. 41 (7), 1757–1762.

[B37] Li L. Zhang C. Xiao Y. Wang Y. Chen D. Tian G. (2011a). Study on simulation processing method for rhubarb pieces. China J. Traditional Chin. Med. Pharm. 26 (8), 1777–1780.

[B38] Li L. Zhang C. Zheng W. Yu Y. Zhang F. Gao H. (2021b). UPLC-QqQ-MS-based quantitative analysis on changes of ten constituents between the raw and salt-water processed Phellodendron chinense. Chin. Tradit. Pat. Med. 43 (11), 3082–3088.

[B39] Li M. Wu S. Gong X. Liu S. hU C. (2017b). Comparison on fingerprint spectrum of urine-reducing part of suoquan wan constituted with non-processed or salt-processed Alpiniae oxyphyllae Fructus fruits. Chin. J. Exp. Traditional Med. Formulae 23 (4), 16–19.

[B40] Li N. Yan D. Zhang J. Gao X. (2017c). Effects of different processing methods on the antiosteoporotic effective compositions of Psoralea corylifolia L. Mod. Traditional Chin. Med. Materia Medica-World Sci. Technol. 19 (1), 127–132.

[B41] Li Q. Sun P. Li J. Pei H. (2019). Effects of salt processing on the contents of gallic acid, ellagic acid and epicatechin in Fructus emblica l. Lishizhen Med. Materia Medica Res. 30 (9), 2155–2158.

[B42] Li S. Peng L. Xia Q. Ao H. Tan R. Wan Z. (2015). Comparison on the chemical constituents and antioxidant activity of volatile oil of Alpiniae oxyphyllae Fructus between the raw and the salt. Inf. Traditional Chin. Med. 32 (6), 23–27.

[B43] Li W. Hu C. Li X. Huang Q. Xie F. Xie X. (2010). Comparison on fingerprints of Fructus Alpiniae oxyphyllae between the raw and the salt by HPLC. Lishizhen Med. Materia Medica Res. 21 (2), 305–306.

[B44] Li X. Yang Z. Shi J. Jiang X. (2011b). Effects of different processing methods on mangiferin content in Anemarrhenae anemarrhenae. Lishizhen Med. Materia Medica Res. 22 (10), 2502–2503.

[B45] Li Z. Sun T. Liu Y. Li Y. Di Z. Liu F. (2021c). Detoxification mechanism of Psoraleae based on analysis of potential hepatotoxic components. J. Chin. Med. Mater. 44 (9), 2091–2094.

[B46] Liang C. Wu S. Wei Y. Jiang Y. Wang D. Yu L. (2017). Effect of raw and salt-processed of psoralen on function of liver and kidney and expression of aquaporins in kidney-yang deficiency model rats. Chin. Traditional Herb. Drugs 48 (22), 4713–4718.

[B47] Liao Y. Ao M. Peng Y. Dong B. Jiang Y. Hu C. (2021a). Effect of salt-water processing on the contents of main chemical constituents in Wuzi Yanzong pills. J. Chin. Med. Mater. 44 (8), 1851–1855.

[B48] Liao Y. Zhang X. Ao M. Hu C. Chen Z. Xu R. (2021b). Effect of herbal medicines in Wuzi Yanzong wan processed with salt-water on apoptosis of spermatogenic cells in rats. Chin. J. Exp. Traditional Med. Formulae 27 (10), 1–7.

[B49] Liu C. (2003). Historical evolution of salt processing of Chinese herbal medicine. Study J. Traditional Chin. Med. 21 (8), 1353.

[B50] Liu F. Xiao F. Li Y. Song X. Chen K. Kan H. (2016a). The optimization of the salt processing procedure of Morinda officinalis How. by uniform design and the comparison of 5 kinds of saccharides's content. J. Guangdong Pharm. Univ. 32 (4), 415–419.

[B51] Liu K. Zhou L. Liu P. Zhang M. Cao Y. Xu N. (2011). Effect of processing with salt·water on the chemical constituents of Eucommiae Cortex. Chin. Tradit. Pat. Med. 33 (2), 280–284.

[B52] Liu S. Zhang S. Wei H. Chen B. Huang Y. Zhong Z. (2018). Preliminary study on educing hepatotoxicity of preparing Euodia rutaecarpa fruit. J. Chin. Med. Mater. 41 (3).

[B53] Liu Y. Yao H. Chen W. Li Q. (2016b). Quantitative analysis of four flavonoids in salt-processed Cuscuta chinensis with different processing methods. China Pharm. 27 (15), 2128–2130.

[B54] Luo W. Phan H. V. Xie M. Hai F. I. Price W. E. Elimelech M. (2017). Osmotic versus conventional membrane bioreactors integrated with reverse osmosis for water reuse: Biological stability, membrane fouling, and contaminant removal. Water Res. 109, 122–134. 10.1016/j.watres.2016.11.036 27883917

[B55] Lv B. Tan W. Liu X. Wang X. Gao X. (2020). Scientific connotation of "Thunder gong processing" of Psoraleae detoxification. Tianjin J. Traditional Chin. Med. 37 (9), 976–983.

[B56] Meng J. Jiang H. Lu J. Xiao Y. Cao L. Ye X. (2017). HS-SPME-GC-MS analysis on volatile components in various processed products of Achyranthes bidentatae Radix. China Pharm. 20 (10), 1745–1748.

[B57] Miao X. Xiao B. Sun Y. Wang C. Yang J. Huang R. (2018). Application of metabolomics in the study of traditional Chinese medicine formula. Mod. Traditional Chin. Med. Materia Medica-World Sci. Technol. 20 (8), 1345–1350.

[B58] Mo S. Zhu H. Liao S. Wen B. (2007). Study on analgesic and anti-inflammatory effects of different medicinal products of Orange kernel. Pharmacol. Clin. Chin. Materia Medica 23 (5), 141–142.

[B59] Pan L. Xu W. Wang N. Xiu Y. (2018). Application of study methods based on animal *in vivo* process in the field of traditional Chinese medicine processing. China J. Traditional Chin. Med. Pharm. 33 (2), 624–627.

[B60] Qian C. Zhu C. Chen Z. (2018). Targeting drug delivery and meridional theory in the processing of Chinese Materia Medica. J. Nanjing Univ. Traditional Chin. Med. 24 (1), 30–24.

[B61] Shao Z. Li L. Wu D. Huang Q. Zhang W. Xu F. (2020). Study on composition changes of flavonoids and saponins in decoction pieces of zhimu anemarrhenae rhizoma before and after processing. J. Shandong Univ. Traditional Chin. Med. 44 (3), 301–308.

[B62] Shuai X. Hu C. Shan S. (2018). The influence of suoquan wan (缩泉丸) formulated by yizhiren (Alpiniae oxyphyllae) processed by salt on serum bun, crea, *etc.* and renal morphology of the kidney yang deficiency model mice. Guid. J. Traditional Chin. Med. Pharm. 24 (13), 15–18.

[B63] Si J. Zhao H. (2019). Effect of salt preparation into kidney on th theory of Chinese medicine property and the clinical use of Chinese medicine. Asia-Pacific Tradit. Med. 15 (9), 158–160.

[B64] Song Z. Wu Y. Gao H. (2015). Determination of neomangiferin, mangiferin, and isomangiferin in Anemarrhenae Rhizoma before and after stir-frying with salt-water by HPLC. Drugs & Clin. 30 (2), 145–148.

[B65] Song Z. Wu Y. Gao H. (2014). Separation and content comparison of vitexin from anemarrhenae rhizoma before and after salt. Process. Chin. J. Inf. Traditional Chin. Med. 21 (12), 79–81.

[B66] Su Z. He Y. Liang H. Jiang X. Wang J. Chen K. (2016). Evaluate the content of chrysin in Fructus Alpiniae oxyphyllae from different producing areas after processed with salt. Chin. J. Ethnomedicine Ethnopharmacy 25 (11), 14–16.

[B67] Sun H. Chen P. Jiao Z. Yue S. Jin T. Xiang L. (2015). Essential oils of Alpiniae Oxyphyllae Fructus before and after salt-fried:GC-MS analysis and their anti-acetylcholinesterase activity. J. Shandong Univ. Heal. Sci. 53 (12), 27–32.

[B68] Tang Y. Xu J. Han X. Han S. Liu K. Zhi M. (2021). Tissue distribution of mangiferin in anemarrhenae rhizoma and salt stir-fried anemarrhenae rhizoma decoction in rats. Mod. Chin. Med. 23 (1), 73–76+93.

[B69] Tao Y. Du Y. Li W. Cai B. (2018). Development and validation of an UHPLC–MS/MS approach for simultaneous quantification of five bioactive saponins in rat plasma: Application to a comparative pharmacokinetic study of aqueous extracts of raw and salt-processed Achyranthes bidentata. J. Pharm. Biomed. 151, 164–169. 10.1016/j.jpba.2017.12.024 29331795

[B70] Tao Y. Huang S. Yan J. Cai B. (2019a). Pharmacokinetic study of six triterpenoids of raw and processed Alisma plantago-aquatica in rat plasma by using ultra performance liquid chromatography-tandem mass spectrometry approach. J. Chromatogr. B 1124, 323–330. 10.1016/j.jchromb.2019.06.026 31271908

[B71] Tao Y. Huang S. Yan J. Li W. Cai B. (2019b). Integrated metallomic and metabolomic profiling of plasma and tissues provides deep insights into the protective effect of raw and salt-processed Achyranthes bidentata Blume extract in ovariectomia rats. J. Ethnopharmacol. 234, 85–95. 10.1016/j.jep.2019.01.033 30784959

[B72] Tao Y. Jiang E. Jiang H. Yan J. Cai B. (2020). Effects of raw and salt-processed Achyranthes bidentata on osteoporosis in zebrafish. J. Zhejiang Univ. Technol. 48 (5), 504–507+525.

[B73] Tao Y. Jiang Y. Li W. Cai B. (2016). Effect of processing on contents of twelve constituents in Psoraleae Fructus. Chin. J. Exp. Traditional Med. Formulae 22 (21), 6–9.

[B74] Wang L. Yan C. Le Z. Dong J. Bai Z. (2017). History evolution of salt production and processing with brine. China J. Chin. Materia Medica 42 (20), 3880–3885. 10.19540/j.cnki.cjcmm.20170928.023 29243421

[B75] Wang X. Jiang Y. Fan L. Wu D. Han Y. Jin C. (2020). Modern research on theory of “salt-processing enhancing drug into kidney meridian” proposed by healer Jia-mo Chen from Xinan. Chin. Traditional Herb. Drugs 51 (5), 1336–1342.

[B76] Wei T. He X. (2020). Advances in processing and quality analysis of Papaya. Chin. J. Clin. Ration. Drug Use 13 (10), 180–181.

[B77] Wei Y. Song C. Wang J. Ma Y. Chen T. (2021). Study on correlation between physical parameters and chemical components of different processed products of huangqin(scutellariae Radix). Chin. Archives Traditional Chin. Med. 39 (5), 119–125.

[B78] Wen X. Song L. He M. (2018). Effect of simple stir-frying and salted stir-frying on the chemical constituents in Alisma Rhizoma by HPLC fingerprints. Glob. Tradit. Chin. Med. 11 (6), 831–836.

[B79] Weng Z. Yan C. Gao Q. Zhao G. Chen Z. Cai B. (2015). Effects of different prepared eucommia ulmoides drug serum and its iridoids on osteoblast proliferation and differentiation in postmenopausal women. Asia-Pacific Tradit. Med. 26 (11), 2636–2638.

[B80] Wu Q. Wang J. Yang A. Zhou N. Zhao Q. Li K. (2021a). To explore synergistic mechanism of Buguzhi Pills based on correlation between antidiarrheal effect and difference of ingredients before and after salt processing. Chin. Traditional Herb. Drugs 52 (21), 6493–6500.

[B81] Wu S. Li M. zheng K. Zhao Y. Hu L. Hu C. (2016a). The characteristic of nootkatone of effective parts from Alpiniae oxyphyllae Fructus after salt processing *in situ* intestinal absorption in rats. Pharmacol. Clin. Chin. Materia Medica 32 (5), 65–68.

[B82] Wu W. Guan L. Wei Y. Jia L. Zeng M. (2021b). Comparative study on synergistic effect of energy metabolism in Morinda officinalis which processing by salt or wine. Clin. J. Traditional Chin. Med. 33 (9), 1750–1753.

[B83] Wu Y. Zhang S. Gao H. (2016b). Evaluation of salt processing on pharmacokinetics of neomangiferin in Anemarrhena rhizoma. Liaoning J. Traditional Chin. Med. 43 (7), 1442–1444.

[B84] Xia Y. Yu L. Wang D. Cui Y. Xiong R. Zhang M. (2016). Study on the effect of deficiency of kidney yang, spleen deficiency of animal model before and after different doses of Psoralea stir-baked with salt solution. Asia-Pacific Tradit. Med. 12 (4), 5–8.

[B85] Xiang Q. Zhao W. Wang C. Wu X. Zhu Y. Yang D. (2021). Analysis of terpenoids in Alismatis rhizoma before and after processing with salt-water based on UPLC-Q-TOF-MS. Chin. J. Exp. Traditional Med. Formulae, 1–14.

[B86] Xiao X. Xu S. Gao Y. Li S. Wu C. Yan S. (2019). Study on chemical constituents of ligustri lucidi Fructus by UPLC-Q/TOF MS. Mod. Food Sci. Technol. 35 (10), 253–260.

[B87] Xiong W. Zhao L. (2020a). Effect effects of salt-processed eucommia on bone metabolism in retinoic acid-induced osteoporosis rats. J. China Prescr. Drug 18 (12), 33–35.

[B88] Xiong W. Zhao L. (2020b). Effect of salt-processed Eucommia ulmoides on Bone Growth and metabolism biochemical indexes in rate with the mechanism of action of osteoporosis. Asia-Pacific Tradit. Med. 16 (12), 23–25.

[B89] Xiong W. Zhao L. (2020c). Effects of salt-processed Eucommia ulmoides on biochemical indices and bone mineral density in rats with retinoic acid-induced osteoporosis. Lishizhen Med. Materia Medica Res. 31 (8), 1866–1867.

[B90] Xu H. (2005). Discussion on the function of salt processing of Traditional Chinese medicine. Chin. J. Inf. Traditional Chin. Med. 22 (5), 74.

[B91] Xu J. Ji F. Hu Z. (2017). Changes of chemical components and quality standards after processing of golden cypress. J. Sichuan Traditional Chin. Med. 35 (10), 138–141.

[B92] Xu N. (2018). Study on processing methods, properties and usage of Traditional Chinese medicine. J. Clin. Med. 5 (29), 197–198.

[B93] Yan D. Gao X. (2020). Effect of processing methods on intestinal absorption of anti-osteoporosis components in Psoralea corylifolia L. Lishizhen Med. Materia Medica Res. 31 (12), 2911–2914.

[B94] Yan L. Wu Y. Gao H. (2019). Determination of 4 kinds of saponnins in rhizoma anemarrhenae before and after processing by HPLC-CAD. Res. Pract. Chin. Med. 33 (1), 46–49.

[B95] Yang J. Lu D. Peng X. Dong L. Shen Y. Li Y. (2018). Effects of Psoralea corylifol L. from different sources and different processing methods on bone formation of osteoblast-like SaOS-2 cells. Hainan Med. J. 29 (15), 2140–2143.

[B96] Yang Y. Zhou Q. Liang Z. (2007). Modern research progress on the processing of Chinese medicine Eucommia ulmoides. J. Jinggangshan Univ. Nat. Sci. 28 (10), 99–102.

[B97] Yu H. Jiang Z. Yang F. Wang Y. (2016). Difference of volatile constituents in fennel and its salted product. Chin. Tradit. Pat. Med. 38 (4), 868–872.

[B98] Zhang C. Wang X. (2003). Application of salt and salt processing in the processing of traditional Chinese medicine. J. Gansu Coll. Traditional Chin. Med. 20 (4), 46–47.

[B99] Zhang F. Meng L. Liu P. Wang X. Wang R. Song C. (2020). Absorption differences between the raw and salt-water processed Phellodendron chinense in rats kidney. Chin. Tradit. Pat. Med. 42 (11), 2954–2959.

[B100] Zhang H. Huang Y. Guo C. Gao G. Wu S. Yang M. (2021a). Study on dynamic changes of pharmacodynamic components of Fructus lucidum salt during processing. Lishizhen Med. Materia Medica Res. 32 (3), 614–618.

[B101] Zhang J. Ju C. Na H. Song Q. (2017a). Study on the historical evolution of traditional Chinese medicine salt making method. Asia-Pacific Tradit. Med. 13 (21), 36–38.

[B102] Zhang J. Wang W. Liu Y. Wang Y. Pei Y. Liu X. (2012). Influence of salt processing on the dissolution of compositions from Phellodendri Amurensis Cortex. J. Shenyang Pharm. Univ. 29 (7), 551–554+559.

[B103] Zhang J. Zhang H. Wang Q. (2017b). Effects of totally depurated polysaccharide in Phellodendron amurense rupr before and after processing on immune function of mice. Liaoning J. Traditional Chin. Med. 44 (6), 1263–1267.

[B104] Zhang L. Shi M. Liu J. Han W. (2018a). Synthesis and pharmacological activities of berberine derivatives: Research advances. J. Int. Pharm. Res. 45 (5), 325–332.

[B105] Zhang S. Liu S. Zhong Z. Chen B. Wei H. (2018b). The relationship of “dose time Toxicity”of different processed products of evodia rutaecarpa (juss.) Benth on hepatotoxicity in mice. Lishizhen Med. Materia Medica Res. 29 (4), 881–884.

[B106] Zhang S. Liu S. Zhong Z. Zeng C. Wu Y. Huang Y. (2017c). Study on hepatotoxicity of different processed products of evodia rutaecarpa (juss) Benth on L02 human normal liver cells *in vitro* . Chin. Archives Traditional Chin. Med. 35 (10), 2664–2667.

[B107] Zhang X. Liao Y. Yang Z. Chen Z. Hu C. (2021b). Effect of Fupenzi(Raspberry) with or without salt processing on kidney improvement in rats with kidney yang deficciency and polyuria. Chin. Archives Traditional Chin. Med. 39 (2), 140–142.

[B108] Zhang Y. Han Y. Hao J. Wang T. Liu E. (2016). Influence of processing time on Eucommia cortex and its pharmacokinetics study. J. Tianjin Univ. Traditional Chin. Med. 35 (5), 322–326.

[B109] Zhang Y. Song S. Peng X. Cui J. (2009). Effects of processing methods on the content of pinolinol diglucoside in Eucommia ulmoides. Asia-Pacific Tradit. Med. 5 (2), 3.

[B110] Zhang Y. Xu H. Xu S. Liu H. Gao Y. Zhang H. (2018c). Study on the influencing factors of stir-heating with salt solution method of semen cuscutae based on multi-index components. China J. Traditional Chin. Med. Pharm. 33 (3), 1110–1113.

[B111] Zhang Z. Hu T. Tian S. Wu J. (2017d). Effect of different salting methods on content of effective components in achyranthis bidentatae adix. Chin. J. Exp. Traditional Med. Formulae 23 (3), 10–13.

[B112] Zhao G. Dai S. Chen R. (2006). Dictionary of traditional Chinese medicine. Shanghai: Shanghai Science and Technology Press.

[B113] Zhao H. Guan S. Wang D. (2021). Effects of different processing methods on ulcerative colitis mice pharmaceutical effects of cortex Phellodendri. World Chin. Med. 16 (4), 608–611.

[B114] Zheng M. Wang X. (2021). Effects of processing methods on contents of two flavonoid glycosides in Amomum Villosum. Lishizhen Med. Materia Medica Res. 32 (3), 623–625.

[B115] Zheng M. Wang X. Xie W. (2021). The effect of processing method on the content of camphor, borneol and bornyl acetate in fujian changtai amomum villosum. Chin. Med. Mod. Distance Educ. China 19 (11), 161–164.

[B116] Zheng W. Yan L. Gao H. (2020). Analysis on changes of chemical composition contents and efficacy of improving insulin resistance in type 2 diabetic mice of anemarrhenae rhizoma before and after processing with salt-water. Chin. J. Exp. Traditional Med. Formulae 26 (22), 140–147.

[B117] Zhou Y. Shi L. Lin S. Xie H. Xia C. Qin K. (2021). Research progress of salt processing mechanism of Traditional Chinese medicine. Chin. Tradit. Pat. Med. 43 (10), 2774–2778.

[B118] Zhu X. Zhou Y. Lu J. Lin S. Xie H. Xia C. (2019). Comparative study on fingerprints of decoctions of processed products of eucommiae cortex. World Chin. Med. 114 (2), 274–282.

