# Supplementary material for: Salt processing: A unique and classic technology for Chinese medicine processing
Source: Front Pharmacol. 2023 Jan 30;14:1116047. doi: 10.3389/fphar.2023.1116047 (PMC9922854; doi:10.3389/fphar.2023.1116047)
Supplement: Supplementary file 1 [file Table1.docx]

**Supplementary material 1. Salt processing TCMs in different periods**

| Periods | Continue to use TCMs | New TCMs |
| --- | --- | --- |
| Southern and Northern Dynasties |  | Coicis Semen, Haliotidis Concha, Ricini Semen, Euodiae Fructus, Chalk, Pheretima, Pig grease, Atractylodis Rhizoma, Ostreae Concha. |
| Sui Dynasty | Haliotidis Concha. | Aconiti Radix, Brassica Rapa. |
| Tang Dynasty |  | Wild Rice Root, *Gleditsia Sinensis* Lam., Armeniacae Semen Amarum, Bark of bark of *Catalpa Ovata* G. Don. |
| Song Dynasty | Euodiae Fructus, Chalk, Haliotidis Concha, Atractylodis Rhizoma, Aconiti Radix, *Gleditsia Sinensis* Lam.. | Cyperi Rhizoma, *Aconitum carmichaeli* Debx., Aconiti Kusnezoffii Radix, Aconiti Lateralis Radix Praeparata, Zingiberis Rhizoma, Foeniculi Fructus, Psoraleae Fructus, Alpiniae Oxyphyllae Fructus, Cuscutae Semen, Drynariae Rhizoma, Penis and testicles of *Callorhimus ursinus* Linnaeus or *Phoca largha* Pallas or *Phoca largha* Pallas, Morindae Officinalis Radix, Citri Reticulatae Pericarpium, Corydalis Rhizoma, Persicae Semen, Achyranthis Bidentatae Radix, [Rhizome](javascript:;) of *Dioscorea Septemloba Thunbt* or *Dioscorea hypoglauca Palibin*, Pharbitidis Semen, Akebiae Caulis, Chaenomelis Fructus, Glycyrrhizae Radix et Rhizoma, Astragaali Radix, Anemarrhenae Rhizoma, Phellodendri Chinensis Cortex, Cicadae Periostracum, Muscovite, Trionycis Carapax, Arctii Fructus, *Hylotelephium erythrostictum* (Miq.) H.Ohba [*Sedum erythrostictum* Miq.], Smilacis Chinae Rhizoma, Curcumae Longae Rhizoma, Ulmus Macrocarpa Hance, Mume Fructus, Arecae Pericarpium, *Myrica Rubra* (Lour.) S. et Zucc., Sulfur, Niter, Cirsii Japonici Herba, Mole Cricket, Terrapin Eggs, Siphonostegiae Herba, Gummy Shark Skin, Mozm, Citri Reticulatae Pericarpium Viride, *Vatica mangachapoi* Blanco, Bombyx Batryticatus, Eucommiae Cortex, Toosendan Fructus, Melanteritum, Arisaematis Rhizoma, Areae Semen. |
| Jin and Yuan Dynasties | Foeniculi Fructus, Euodiae Fructus, Aconiti Kusnezoffii Radix, Aconiti Lateralis Radix Praeparata, Cuscutae Semen, Atractylodis Rhizoma, Pharbitidis Semen, Anemarrhenae Rhizoma, Phellodendri Chinensis Cortex, Astragaali Radix, Eucommiae Cortex, Toosendan Fructus, Ostreae Concha, Cyperi Rhizoma, Citri Reticulatae Pericarpium, Alpiniae Oxyphyllae Fructus Morindae Officinalis Radix, *Gleditsia Sinensis* Lam., Smilacis Chinae Rhizoma, | Piperis Longi Fructus, Artemisiae Argyi Folium, Trigonellae Semen, Magnoliae Officinalis Cortex, Citri Exocarpium Rubrum, Zingiberis Rhizoma Recens, Angelicae Dahuricae Radix, Rehmanniae Radix, Melo Semen, Hirudo, Sappan Lignum, *Vatica mangachapoi* Blanco, Selaginellae Herba, Acori Tatainowii Rhizoma, Paeoniae Radix Alba, Platycladi Cacumen. |
| Ming Dynasty | Aconiti Radix, Aconiti Kusnezoffii Radix, Aconiti Lateralis Radix Praeparata, Artemisiae Argyi Folium, Euodiae Fructus, Foeniculi Fructus, Eucommiae Cortex, Psoraleae Fructus, Alpiniae Oxyphyllae Fructus, Morindae Officinalis Radix, Magnoliae Officinalis Cortex, Atractylodis Rhizoma, Citri Reticulatae Pericarpium, Citri Exocarpium Rubrum, Toosendan Fructus, Cyperi Rhizoma, Corydalis Rhizoma, Hirudo, Anemarrhenae Rhizoma, Phellodendri Chinensis Cortex, Haliotidis Concha, Glycyrrhizae Radix et Rhizoma, Astragaali Radix, Brassica Rapa, Coicis Semen, Pharbitidis Semen, Muscovite, Selaginellae Herba, Rehmanniae Radix, Mole Cricket, Sappan Lignum, Arisaematis Rhizoma, Ulmus macrocarpa Hance, Gummy Shark Skin, Pheretima, Piperis Longi Fructus, Rhizoma, Paeoniae Radix Alba, Platycladi Cacumen, Ricini Semen, Ostreae Concha, Arecae Semen, *Aconitum carmichaeli* Debx., Mume Fructus, Angelicae Dahuricae Radix, Chalk, Brassica rapa, *Gleditsia Sinensis* Lam., Corydalis Rhizoma, Melanteritum. | Alpiniae Officinarum Rhizoma, Anisi Stellati Fructus, Serpentis Periostracum, Cervi Cornu Pantotrichum, Chuanxiong Rhizoma, Scorpio, Angelicae Sinensis Radix, Pinelliae Rhizoma, Ophiopogonis Radix, Asparagi Radix, Mantidis Ootheca, Myristicae Semen, Angelicae Pubescentis Radix, Ginseng Radix et Rhizoma, Dioscoreae Rhizoma, Atractylodis Macrocephalae Rhizoma, Nelumbinis Semen, Gardeniae Fructus, Coptidis Rhizoma, Cimicifugae Rhizoma, Serpentis Periostracum, Gypsum、Talcum, Calcite, Magnetitum, Sichuan Pepper, Bark of *Syzygium aromaticum* (L.) Merr.et Perry, Shrimp、Gecko, Lycii Fructus, Peanut, Litsea Pungens, *Luffa cylindrica* (L.) Roem., Nelumbinis Stamen, Violae Herba, Cynanchi Atratii Radix et Rhizoma, *Asparagus cochinchinensis*（Lour.）Merr., Carthami Flos, Realgar, Polygalae Radix, Waterelm, Stellaria media (L.) Cyr., European Heliotrope, Horsebean, Trachycarpi Petiolus, Polygonum Chinense, *Ficus carica* L., Cleome gynandra, Menyanthes Trifoliata, Photinia glabra (Thunb.) Maxim. Crataegus glabr Thunb., Mori Cortex, Cinnabaris, Excrementum Bombycis, Semen Nelumbinis, Herba Eupatorii, Allii Sativi Bulbus, Phytolaccae Radix, Lilii Bulbus, Prunus Persica, Leaf of Xanthium sibiricum Patr., Pteris vittate, *Hyoscyamus Niger*, Argentum, Hydrargyrum, *Cirsiumsetosum*（Willd.）MB., Littleleaf Lemmaphyllum, Fistular Onion Leaf, Nidus Vespae, *Lysimachia Foenum-graecum* Hance, Leaf of *Ulmus pumila* L., Heart of *Capra hircus* Linnaeus or *Ovis aries* Linnaeus, Sheep Kidney, Aurum, Plumbum, Root of *Rosa multiflora* Thunb., Citri Reticulatae semen, Larva of *Anoplophora chinensis* Forster. or *Apriona germari* (Hope), Rhapontici Radix, Auricularia auricula (L.ex Hook.) Underwood, Herba Ecliptae, Raphanus sativus L., *Lactuca sativa* Linn., Stachys sieboldii, Rhei Radix et Rhizoma, Cinnamomum cassia Nees ex Blume, Phyllanthi Fructus, Fruit of *Ilex purpurea* Hassk., Ripe Fruit of *Setaria italica(L.)Beauv.*, *Alpinia japonica* (Thunb.) Miq., *Parthenocissus tricuspidata* (Sieb. & Zucc.) Planch., Ajugae Herba, Asteris Radix et Rhizoma, Amyda sinensis (Wiegmann), Abemoschi Corolla. |
| Qing Dynasty | Aconiti Lateralis Radix Praeparata, Euodiae Fructus, Foeniculi Fructus, Anisi Stellati Fructus, Eucommiae Cortex, Psoraleae Fructus, Cuscutae Semen, Alpiniae Oxyphyllae Fructus, Ginseng Radix et Rhizoma, Dioscoreae Rhizoma, Astragaali Radix, Cyperi Rhizoma, Citri Reticulatae Pericarpium, Toosendan Fructus, Phellodendri Chinensis Cortex, Nelumbinis Semen, Coicis Semen, [Rhizome](javascript:;) of *Dioscorea septemloba Thunbt* or *Dioscorea hypoglauca Palibin*, Pharbitidis Semen, Corydalis Rhizoma, Pheretima, Achyranthis Bidentatae Radix, Rehmanniae Radix, Pinelliae Rhizoma, Muscovite, Mole Cricket, Citri Reticulatae semen, Herba Ecliptae, Mume Fructus, Polygalae Radix, Gardeniae Fructus, Anemarrhenae Rhizoma, Angelicae Dahuricae Radix, Toosendan Fructus, Atractylodis Rhizoma, Cinnabaris, Ricini Semen, Ostreae Concha, Chuanxiong Rhizoma, *Asparagus cochinchinensis* (Lour.) Merr., Armeniacae Semen Amarum, excrementum bombycis, *Lysimachia Foenum-graecum* Hance. | Zanthoxyli Pericarpium, Piperis Fructus, Ligustri Lucidi Fructus, Dendrobii Caulis, Schisandrae Chinensis Fructus, Corni Fructus, Astragali Complanati Semen, Alismatis Rhizoma, Plantaginis Semen, *Euphorbia pekinensis* Rupr., Litchi Semen, Amomum villosum, Aurantii Fructus, Vespae Nidus, Sojae Semen Nigrum, Salviae Miltiorrhizae Radix et Rhizoma, Wall Lizard, Asiatic Bilberry, Eriobotryae Folium, Galla Chinensis, *Bullacta exarate* (Philippi). |
| Modern | Morindae Officinalis Radix, Astragali Complanati Semen, Psoraleae Fructus, Cuscutae Semen, Trigonellae Semen, Eucommiae Cortex, Alpiniae Oxyphyllae Fructus, Cyperi Rhizoma, Amomum villosum, Aurantii Fructus, Toosendan Fructus, Litchi Semen, Citri Reticulatae semen, Citri Reticulatae Pericarpium, Anemarrhenae Rhizoma, Coptidis Rhizoma, Phellodendri Chinensis Cortex, Gardeniae Fructus, Haliotidis Concha, Zingiberis Rhizoma, Euodiae Fructus, Zanthoxyli Pericarpium, Foeniculi Fructus, Atractylodis Rhizoma, Atractylodis Macrocephalae Rhizoma, Alismatis Rhizoma, Plantaginis Semen, Cyathulae Radix, Achyranthis Bidentatae Radix, Paeoniae Radix Alba, Mantidis Ootheca, Corni Fructus, Schisandrae Chinensis Fructus, Ligustri Lucidi Fructus, Lycii Fructus, Platycladi Cacumen, Astragaali Radix, Rhei Radix et Rhizoma. | Dipsaci Radix, Cibotii Rhizoma, Cynomorii Herba, Rosae Laevigatae Fructus, Euryales Semen, Rubi Fructus, Abutili Semen, Scrophulariae Radix, Imperatae Rhizoma, Linderae Radix, Sanguisorbae Radix, Sophorae Flos, Alpiniae Katsumadai Semen, Puncturevine Caltrop Fruit, *Oroxylum indicum* (Linn.) Kurz, Arabic Cowry Shell, Ziziphi Spinosae Semen, Raphani Semen, Allii Tuberosi Semen, Cistanches Herba, Phellodendri Amurensis Cortex, Eucommiae Folium, Tribuli Fructus. |
